# Clinical efficacy of non-pharmacological treatment of functional constipation: a systematic review and network meta-analysis

**DOI:** 10.3389/fcimb.2025.1565801

**Published:** 2025-05-29

**Authors:** Shufa Tan, Chengtao Peng, Xin Lin, Chuanyue Peng, Yunyi Yang, Shuang Liu, Ling Huang, Yuhong Bian, Yuwei Li, Chen Xu

**Affiliations:** ^1^ School of Intergrative Medicine, Tianjin University of Traditional Chinese Medicine, Tianjin, China; ^2^ The First Affiliated Hospital, Jiangxi Medical College, Nanchang University, Nanchang, China; ^3^ Department of Anorectal, Tianjin Union Medical Center The First Affiliated Hospital of Nankai University, Tianjin, China; ^4^ Shanghai University of Traditional Chinese Medicine, Shanghai, China; ^5^ College of Chemistry, Nankai University, Tianjin, China

**Keywords:** network meta-analysis, functional constipation, gut microbiota, chemical drugs, fecal microbiota transplantation, clinical efficacy

## Abstract

**Introduction:**

The purpose of this study is to compare the relative effectiveness and safety of non-pharmacological interventions for the treatment of functional constipation (FC).

**Methods:**

We searched Pubmed, Embase, Cochrane, and Web of Science databases for randomized controlled trials published from 2010 to November 2024. The quality of the included studies was evaluated using the Cochrane bias risk tool and Review Manager 5.4, and the evidence was graded using GRADEPro. A network meta-analysis (NMA) was conducted using R Studio, and the surface under the cumulative ranking curve (SUCRA) was used to rank the included drugs for each outcome measure to compare the clinical efficacy of different treatment methods for chronic functional constipation.

**Results:**

A total of 29 RCT studies were included, with a total of 4389 patients with functional constipation who were randomly assigned to receive placebo or one of the nine different non-pharmacological treatment methods. The assessment of bias risk showed that the bias risk of most included studies was low. The results showed that the first-ranked treatment method for clinical efficacy was acupuncture; the first-ranked treatment method for changes in spontaneous bowel movement (SBM) and complete spontaneous bowel movement (CSBM) was fecal microbiota transplantation (FMT); the first-ranked treatment method for changes in the Bristol Stool Form Scale (BSFS) score was FMT; the first-ranked treatment method for changes in the Patient Assessment of Constipation Quality of Life (PAC-QOL) score after treatment was the Vibration capsule; the first-ranked treatment method for changes in the Patient Assessment of Constipation Symptoms (PAC-SYM) score after treatment was percutaneous electrical stimulation; and the treatment method with the lowest incidence of adverse reactions was probiotics.

**Conclusion:**

Based on the SUCRA values and NMA results, we found that FMT showed better effects and higher safety on BSFS scores, SBM, and CSBM. In addition, acupuncture showed a good clinical efficacy. We hypothesize that the combination of FMT and acupuncture may be an effective and safe treatment option for functional constipation, but further high-quality clinical studies are needed to confirm this.

**Systematic review registration:**

https://www.crd.york.ac.uk/prospero/, identifier CRD42024625747.

## 1 Introduction

Unlike constipation caused by organic etiology, physiological structural abnormalities, and metabolic disorders, FC is a disorder of gastrointestinal dynamics resulting from brain-gut interactions and affects approximately 14% of the global population (Vriesman et al., 2020).The pathogenesis of FC involves several factors, including poor dietary habits, reduced gastrointestinal motility, abnormal mucosal immunoregulation, visceral hypersensitivity, altered intestinal microbiota, and changes in the central nervous system. Clinically, FC is characterized by infrequent defecation (fewer than three times per week) over a three-month period, accompanied by difficult defecation, dry and hard stools, and a sensation of incomplete evacuation, all occurring in the absence of underlying organic pathology (Aziz et al., 2020; Barberio et al., 2021). Chronic constipation not only imposes significant costs on the healthcare system, but it also negatively impacts patients’ quality of life. Individuals suffering from constipation frequently experience psychological and emotional distress, which can be exacerbated by adverse mental health states, thereby intensifying the severity of their symptoms (Wang et al., 2023); Moreover, chronic constipation can lead to serious complications such as fecal perforation of the colon, cardiovascular and cerebrovascular events, and colorectal tumors, posing indirect threats to life (Zheng et al., 2024; Staller et al., 2022). Constipation is considered to be an independent risk factor for cardiovascular disease death. When defecating hard, blood pressure rises transiently, which may induce angina pectoris, myocardial infarction, and cerebral hemorrhage (Park et al., 2025).In China, the treatment of FC primarily involves dietary modifications, changes in defecation habits, the use of anal plugs, enemas, oral medications, surgery, and biofeedback therapy. These approaches can promote defecation and alleviate symptoms to some extent (Serra et al., 2020a).Stimulant laxatives are commonly employed, providing rapid relief from constipation. However, prolonged use of laxatives may disrupt the intestinal microbiota. Similarly, the use of corkscrews and enemas can result in abdominal pain, and long-term reliance on these treatments often leads to poor symptomatic relief, secondary habitual constipation, and potential damage to intestinal structure and function, ultimately exacerbating constipation (Cui et al., 2020).Surgical treatment for chronic constipation is more invasive and carries a risk of postoperative complications. While medications can provide temporary relief, their long-term use may damage intestinal nerve endings, leading to drug dependence, tolerance, and adverse reactions. These issues can exacerbate constipation and contribute to complications such as colorectal degeneration following the discontinuation of the medication. Most conventional laxatives alleviate symptoms by temporarily increasing intestinal water content or stimulating bowel motility, yet fail to correct underlying pathophysiological abnormalities. Prolonged use often compels dose escalation or drug substitution, thereby exacerbating recurrence risks. Studies indicate that nearly 50% of patients discontinue medication prematurely due to inconsistent efficacy or adverse effects, while coexisting unhealthy lifestyle habits further compromise therapeutic outcomes (Rao and Brenner, 2021). As a result, clinicians are increasingly focusing on non-pharmacological treatments. Several global guidelines now recognize non-pharmacological interventions as the first-line approach for managing constipation (Functional Gastrointestinal Disease Collaborative Group et al., 2024; Chang, 2024; Alavi et al., 2024).Conventional non-pharmacological treatments for constipation include dietary modifications, acupuncture, transcutaneous electrical stimulation, and massage. Acupuncture has been utilized for approximately 3,000 years in China to address gastrointestinal symptoms (Liu et al., 2020). The World Health Organization (WHO) acknowledges constipation as one of the indications for acupuncture, recommending it as a viable therapeutic approach (Wang et al., 2023), Acupuncture is believed to regulate gastrointestinal motility and gastric acid secretion while influencing gastrointestinal cells, hormones, and neurotransmitters within the enteric nervous system, thereby enhancing gastrointestinal function and alleviating constipation (Tan et al., 2024). Studies have demonstrated differences in the species composition and abundance of fecal microorganisms between patients with constipation and those with normal intestinal function, although the findings remain inconsistent. It is evident, however, that constipation is frequently associated with dysbiosis, which is characterized by a decrease in beneficial microbial strains and an increase in harmful ones (Liu et al., 2022). Consequently, therapeutic strategies aimed at modulating the intestinal microbiota—such as the use of probiotics, prebiotics, and fecal transplants—have emerged as a significant focus of contemporary research. Non-pharmacological interventions, which can prevent constipation and alleviate patients’ discomfort without the side effects associated with medications, play a vital role in enhancing both the quality of life and the symptoms experienced by those affected by constipation (Staller and Cash, 2020). Nevertheless, uncertainty persists regarding the balance of benefits and drawbacks among these interventions, owing to the existence of a wide array of treatment modalities.

Bayesian NMA is an advanced form of traditional meta-analysis that incorporates indirect comparisons of interventions, thereby facilitating the assessment of the relative effects of three or more treatments. This approach allows for the ranking and hierarchical comparison of interventions. Although non-pharmacological interventions such as dietary fiber supplementation, probiotics, FMT, exercise therapy, abdominal massage, biofeedback training, and acupuncture are widely recommended, consensus remains lacking regarding their relative efficacy and optimal combinations. This study aims to systematically evaluate the differences in efficacy and safety of non-pharmacological interventions for chronic constipation patients through a NMA, addressing the current limitations of fragmented evidence and insufficient head-to-head comparative studies. By quantifying intervention prioritization via the surface under the cumulative ranking curve, we will resolve ambiguities in therapeutic hierarchies. Additionally, subgroup analyses stratified by patient characteristics (age, constipation severity) will elucidate sources of heterogeneity and refine precision in clinical decision-making. The findings are expected to provide evidence-based guidance for selecting non-pharmacological therapies and bridge critical evidence gaps in guideline recommendations regarding intervention intensity and prioritization.

## 2 Methods

### 2.1 Study design

This study was previously registered with the International Registry for Prospective Systematic Reviews (PROSPERO) (registration number: CRD42024625747).

### 2.2 Search strategy

Two researchers independently searched PubMed, Web of Science, Embase, and the Cochrane database using terms including chronic constipation, constipation, and functional constipation (using the Mesh term function in PubMed to expand the search scope). Fecal microbiota transplantation, FMT; Electroacupuncture, acupuncture; Vibration capsule; Percutaneous electrical stimulation; Probiotics; Prebiotics; Fibers; Dietary therapy; Non-drug intervention. Search fields include keywords, titles, and abstracts, with a search strategy limited to RCT studies, without language or time restrictions. The search time range is from January 2010 to November 1, 2024. The search strategy is as follows: ((chronic constipation) OR (constipation)) OR (functional constipation)) AND (((((((((((Fecal microbiota transplantation) OR (FMT)) OR (Electroacupuncture)) OR (Acupuncture)) OR (Vibration capsule)) OR (Percutaneous electrical stimulation)) OR (Probiotics)) OR (Prebiotics)) OR (Fibers)) OR (Dietary therapy)) OR (Non-drug intervention)). Filters: Randomized Controlled Trial, from 2010 - 2024. The systematic review will be conducted according to the Preferred Reporting Items for Systematic Reviews and Meta-Analyses (PRISMA) guidelines for systematic reviews and meta-analyses (Page et al., 2021).

### 2.3 Inclusion and exclusion criteria

Inclusion criteria:

1. Subjects were patients who met the Rome IV diagnostic criteria for functional constipation and were confirmed by direct anal manometry, balloon discharge test, and colon transport test: After the exclusion of organic lesions, two or more of the following symptoms had to be included: ①At least 25% effortlessness in bowel movements; ② At least 25% of bowel movements were hard and dry masses (Bristol stool type 1 or 2);③ At least 25% of the defecation had incomplete feeling (the feeling of having not finished the stool after defecation);④ At least 25% of defecation had a sense of anorectal obstruction;⑥ At least 25% of defecation needs manual assistance;⑦ Spontaneous bowel movements less than 3 times per week);
2. The study type was a randomized controlled trial;
3. The study subjects included patients with functional constipation who used different non-pharmacological intervention methods, including probiotics, prebiotics, acupuncture, transcutaneous electrical nerve stimulation, biofeedback, fecal microbiota transplantation, vibrating capsule, dietary fiber, and dietary interventions;
4. Both Chinese and English literature.

Exclusion criteria:

1. Literature could not be found or data were missing and could not be used for statistical analysis;
2. Duplicate publications;
3. *In vitro* experiments, animal experiments, non-comparative studies, meta-analyses, reviews, letters, guidelines, case reports, etc;
4. Literature quality was low or the sample size included was too small(Studies with fewer than 10 patients were excluded);

### 2.4 Literature screening and data extraction

Literature obtained from the four database searches was imported into EndNote, the literature management software, and subsequently reviewed by two authors based on the titles and abstracts of the articles. Decisions regarding the inclusion or exclusion of studies were made by consensus, adhering to the predefined criteria. Information related to the included literature, study objectives, outcomes, and follow-up data was independently extracted using a standardized data extraction form and was cross-checked by two researchers. Any disagreements were resolved through discussion. For each study, the following information was collected: (1) study characteristics, including the first author, country, year of publication, type of study, and trial protocol; (2) patient baseline data, comprising intervention modality, number of patients, age, and number of females; and (3) study outcomes, where primary outcome indicators included clinical effectiveness (defined as the proportion of patients with ≥3 CSBMs per week), BSFS scores, SBM change values, and CSBM change values. Secondary outcome indicators included the adverse event rate, PAC-SYM score change values, and PAC-QOL score change values.

### 2.5 Risk of bias assessments

The risk of bias of the included literature was assessed by 2 researchers in strict accordance with the Cochrane Risk of Bias Assessment Tool and analyzed using Review Manager 5.4:7 entries were included: random allocation, allocation concealment, blinding of subjects and implementers to interventions, blinding of outcomes, outcome evaluation, completeness of outcome data, selection of studies to report findings, and other bias, each of which was assessed for risk of bias in terms of Each entry was assessed for risk of bias according to “low risk”, “unknown risk”, and “high risk”. GRADEPro was used to evaluate the level of evidence. If the difference between the two assessments was large or affected the inclusion of the study in the final analysis, a third-party expert was consulted to resolve the issue.

### 2.6 Statistical analysis

R Studio was used to call gemtc and BUGSnet software packages to establish Bayesian models for network meta-analysis, draw probability maps and perform probability ranking. Relative risks (RR) and 95% confidence intervals (CI) were calculated for dichotomous variables, and standardized mean deviations (WMD) and 95% CIs were calculated for continuous variables. When a closed loop was formed between interventions, an inconsistency test was required to assess the degree of agreement between the results of direct and indirect comparisons, and the test of inconsistency was performed using nodal analysis. if P>0.05 then no difference was indicated. The overall ranking of treatments was estimated by calculating the SUCRA value for each method, and the interventions were ranked as superior or inferior according to the magnitude of the SUCRA value.

## 3 Results

### 3.1 Literature search results

**Figure 1:**
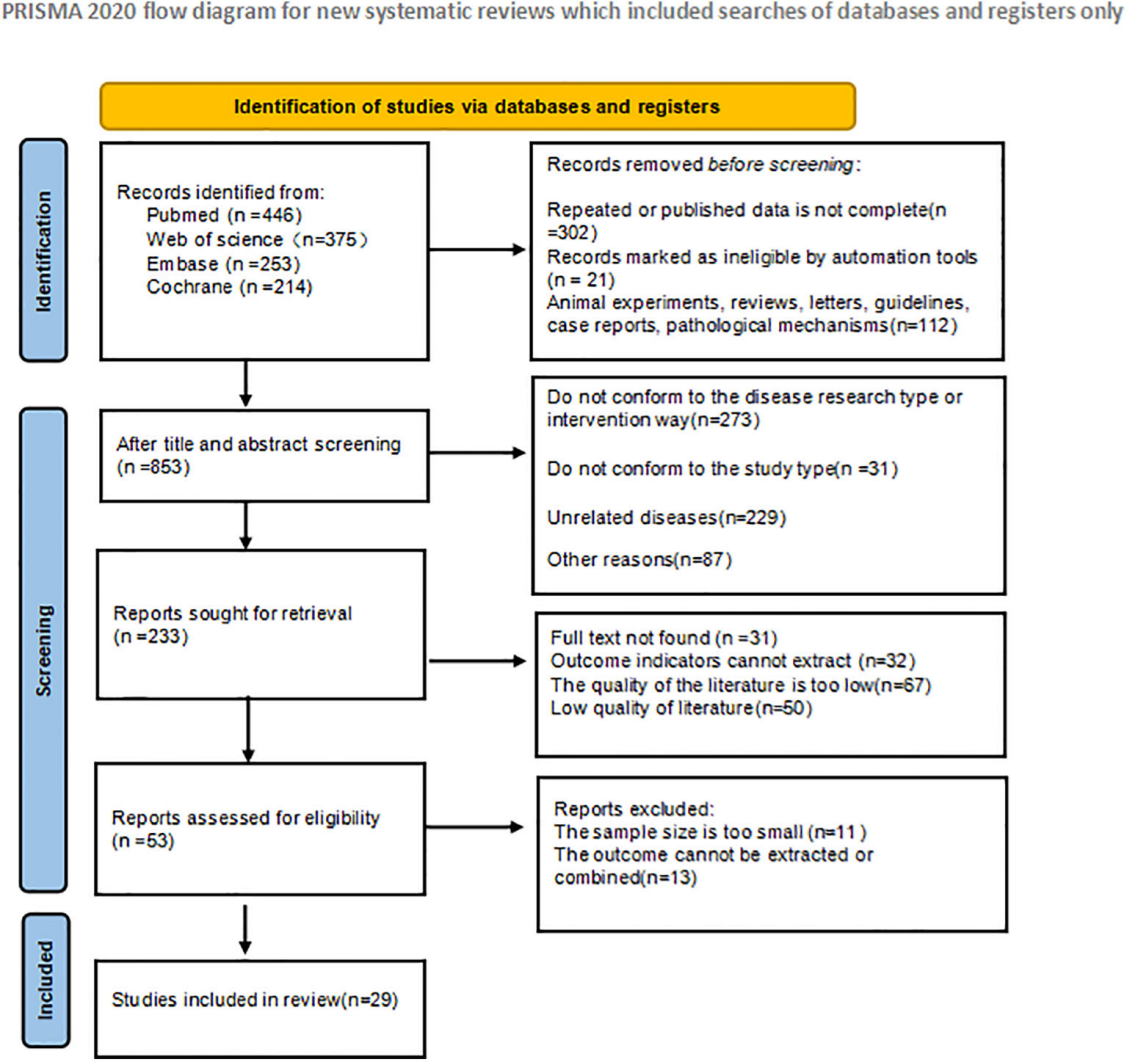
Literature screening process.

A total of 1288 articles were retrieved in the initial literature search. Duplicate studies as well as ineligible studies were excluded 435; after reading the article titles and abstracts, 800 were excluded based on nerfing criteria and 53 studies were initially included. Subsequently, we read the full text and excluded 14 studies that did not meet the inclusion criteria. Twenty-nine RCT studies (Mazlyn et al., 2013; Dupont et al., 2014; Liu et al., 2016; Tian et al., 2017; Lee et al., 2018; Riezzo et al., 2018; Yoon et al., 2018; Bilgili, 2019; Dimidi et al., 2019; Ibarra et al., 2019; Zhou et al., 2019; Maeta et al., 2020; Moore et al., 2020; Rao et al., 2020; Bayer et al., 2022; Chan et al., 2022; Doğan et al., 2022; Wang et al., 2022; Zeng and Chen, 2022; Zhu et al., 2022; Aminizadeh et al., 2023; Lai et al., 2023; Ma et al., 2023; Rao et al., 2023; Takeda et al., 2023; Tierney et al., 2023; Gu et al., 2024; Salo et al., 2024; Yang et al., 2024a) were finally included for net Meta-analysis, and the literature screening process and results are shown in **Figure 1**.

### 3.2 Basic characteristics of the included studies

As shown in **Table 1**, the 29 included studies were RCTs with a total of 4,389 patients with functional constipation, including 2,197 in the experimental group and 2,192 in the control group. The study characteristics, patient baseline, and study outcomes of the included studies are displayed in **Table 1**.

**Table 1:** Clinical and Demographic Characteristics of Studies Included in NMA.

Author	Year	Country	Study Type	Intervention Mode		Number of Cases		Age years		Females		Inclusion criteria	Concrete way	Outcome Indicators
				Experimental group	Control Group	Experimental group	Control Group	Experimental group	Control Group	Experimental group	Control Group			
E. Salo	2024	Spain	RCT	Probiotics	Placebo	23	23	43.2(15.6)	53.0(13.9)	20	22	Constipation that lasted more than three months, with less than three bowel movements per week, and/or Bris-tol Scale 1 and 2.	Bifidobacteriumlactis BLa80	1
Dimidi, E	2019	UK	RCT	Probiotics	Placebo	37	38	35(12)	31(10)	34	35	Self-reported stool frequency of 3 or less bowel movements per week; stool consistency of type 1-4 on the Bristol Stool Form Scale;fulfilment of modified Rome III diagnostic criteria for functional constipation	B.lactis NCC2818	2、3、4、5、6
Teng Ma	2023	China	RCT	Probiotics	Placebo	78	85	22.68 ± 5.66	21.59 ± 4.59	63	68	Rome IV criteria for functional constipation based on self-reporting over the past three months, with symptom onset within the last six months	Lactiplantibacillus plantarum P9	2、3、4
Tsutomu Takeda	2022	Japan	RCT	Probiotics	Placebo	39	41	78.1±6.4	77.9±6.2	18	18	Patients diagnosed with functional constipation according to the Rome IV diagnostic criteria	Probiotic (BB536)	3
Luyao Wang	2022	China	RCT	Probiotics	Placebo	53	50	51.7 (12.8)	47.6 (13.6)	42	46	Eligible patients were Chinese adults aged 18–80 years who satisfied the Rome IV diagnostic criteria	B. bifidum CCFM16	1、3、4、5、6
Braden T. Tierney	2020	USA	RCT	Probiotics	Placebo	33	31	7.79 (3.25)	9.84 (3.87)	12	15	Patients diagnosed with functional constipation according to the Rome IV diagnostic criteria	synbiotic	1
Hao Lai	2023	China	RCT	Probiotics	Fibers	50	50	43.1±15.6	46.9±17.4	40	41		lactis HN019 + Lacticaseibacillus rhamnosus HN001	4
Hao Lai	2023	China	RCT	Probiotics	Placebo	50	50	43.1±15.6	46.9±17.4	40	38			
G.Riezzo	2018	Italy	RCT	Probiotics	Placebo	28	28	42.1±11.6	45.5±11.3	28	28	Fulfilment of the Rome III Criteria for FC without matching Rome criteria	Lactobacillus reuteri DSM 17938	4
Jin Young Yoon	2018	Korea	RCT	Probiotics	Placebo	88	83	38.3 ± 8.9	39.4 ± 11.4	74	73	Fulfilment of the Rome III Criteria for FC without matching Rome criteria	Streptococcus thermophilus MG510	7
Mena Mustapha Mazlyn	2013	Malaysia	RCT	Probiotics	Placebo	47	43	31.8± 9.4	31.7 ± 9.4	42	36	Diagnosed with Rome II-defined functional constipation with CCQ score of at least 5	Shirota fermented milk	3、4
Christophe Dupont	2014	France	RCT	Diet	Placebo	85	77	44.4±10.5	40.8±12.4	85	77	Diagnosis of constipation according to the Rome III criteria20 since at least the past 3 months	Waters, Issy-les-Moulineaux, France	1、5
Simone B. Bayer	2022	New Zealand	RCT	Diet	Fibers	11	32	40.2 ± 4.1	35.0 ± 2.7	10	26	Fulfilment of the Rome III Criteria for FC without matching Rome criteria for IBS	Gold kiwifruit (two daily)	2、4
Akihiro Maeta	2020	Japan	RCT	Diet	Placebo	25	25	21 (20–21)	21 (20–21)	25	25	Constipation according to the diagnostic criteria of Rome IV	okara soup	3
Satish S.C	2023	USA	RCT	Vibration capsule	Placebo	163	149	47.1 (13.33)	45.9 (13.47)	143	126	Adults (≥22 years) with chronic idiopathic constipation according to Rome III criteria	Vibrating Capsule	1、2、3、4、5、7
Satish S. C. Rao	2020	USA	RCT	Vibration capsule	Placebo	89	93	45.36 ± 13.08	42.67 ± 11.15	71	71	All subjects were adults ≥ 22 years old who had between 1 and 3 sponta-neous bowel movements (SBM) per week.	Vibrating Capsule	2
Jia-Hui Zhu	2022	China	RCT	Vibration capsule	Placebo	53	53	42.8 (14.3)	43.2 (13.4)	48	48	Self-reported com-plete spontaneous bowel movements (CSBMs) per week as less than three for the last three months	Vibrating Capsule	1、2、3、5、6
Jingze Yang	2024	China	RCT	Percutaneous electrical stimulation	Placebo	35	35	34.91±11.37	37.14±13.02	32	31	Inclusion criteria included 1) <three spontaneous bowel movements (SBMs) per week; and 2) <80% of radiopaque marker excretion after 72 hours in a colon transit test	Transcutaneous electrical acustimulation	3、5、6
Yuxiao Zeng	2022	China	RCT	Percutaneous electrical stimulation	Electroacupuncture	35	35	46.60 (17.32)	45.26 (14.22)	26	29	With functional constipation as defined by the Rome IV criteria1; with 2 or fewer complete sponta-neous bowel movements (CSBMs) per week	Electroacupuncture	2、3、4、7
Judith S. Moore	2019	Australia	RCT	Electroacupuncture	Placebo	17	16	45 (19-67)	44 (23-66)	17	16	Constipation according to the diagnostic criteria of Rome IV	Electroacupuncture	1、5、6
Jing Zhou	2019	China	RCT	Electroacupuncture	Placebo	415	407	45.60 (15.69)	45.49 (15.11)	415	407	Constipation according to the diagnostic criteria of Rome IV	Electroacupuncture	1、2、3、4、5、7
Hye-Yoon Lee	2018	Korean	RCT	Electroacupuncture	Placebo	14	15	49.6 ± 12.7	50.0 ± 10.5	12	14	Constipation according to the diagnostic criteria of Rome IV	Electroacupuncture	2、4、7
Zhishun Liu	2016	China	RCT	Electroacupuncture	Placebo	536	539	47.01 (16.5)	47.33 (15.8)	415	407	Based on the Rome III diag-nostic criteria for functional gastrointestinal disorders, had CSFC with 2 or fewer mean weekly CSBMs for more than 3 months	Electroacupuncture	1、2、3、5、7
Xu GU	2024	China	RCT	FMT	Placebo	55	55	6.32±2.24	6.76±2.90	25	24	Patients met the Rome IV criteria for childhood constipation	FMT	1、3、7
Hongliang Tian	2017	China	RCT	FMT	Placebo	30	30	53.1±10.2	55.4±12.1	30	30	Patients who were diagnosed as STC and were not responsive to traditional treatments with diet modification	FMT	1、2、4
Mahdi Aminizadeh	2023	Iran	RCT	Massage	Placebo	30	30	74.20 ± 5.37	72.77 ± 4.20	16	16	Constipation according to the diagnostic criteria of Rome IV	Auricular acupressure	5、6
Irem Gül Dogan	2022	Turkey	RCT	Massage	Placebo	37	37	40.1 (11.3)	36.4 (11.6)	30	30	Constipation according to the diagnostic criteria of Rome IV	Abdominal Massage	4、5
Canan Birimoglu	2019	Turkey	RCT	Massage	Placebo	17	18	80.6±6.8	78.2±6.3	10	9	Diagnosed constipation according to Rome II Diagnostic Criteria for Constipation	Abdominal Massage	1
T.C. Chan	2022	China	RCT	Fibers	Placebo	26	26	82.7±7.8	84.4±6.9	16	13	They had constipation fulfilling Rome III criteria for functional constipation Fewer than three defecations per week	Fibers	4、7
Alvin Ibarra	2019	Canada	RCT	Fibers	Placebo	48	48	41.5±17.1	43.6±13.4	32	33	Less than 3 bowel movements per week	Fibers	4、5、6

(1: Overall response rate 2: CSBM change 3: SBM change 4: BSFS 5:PAC-QOL 6:PAC-SYM 7:Adverse event NA: Unable to extract).

### 3.3 The quality assessment of the included studies

**Figure 2:**
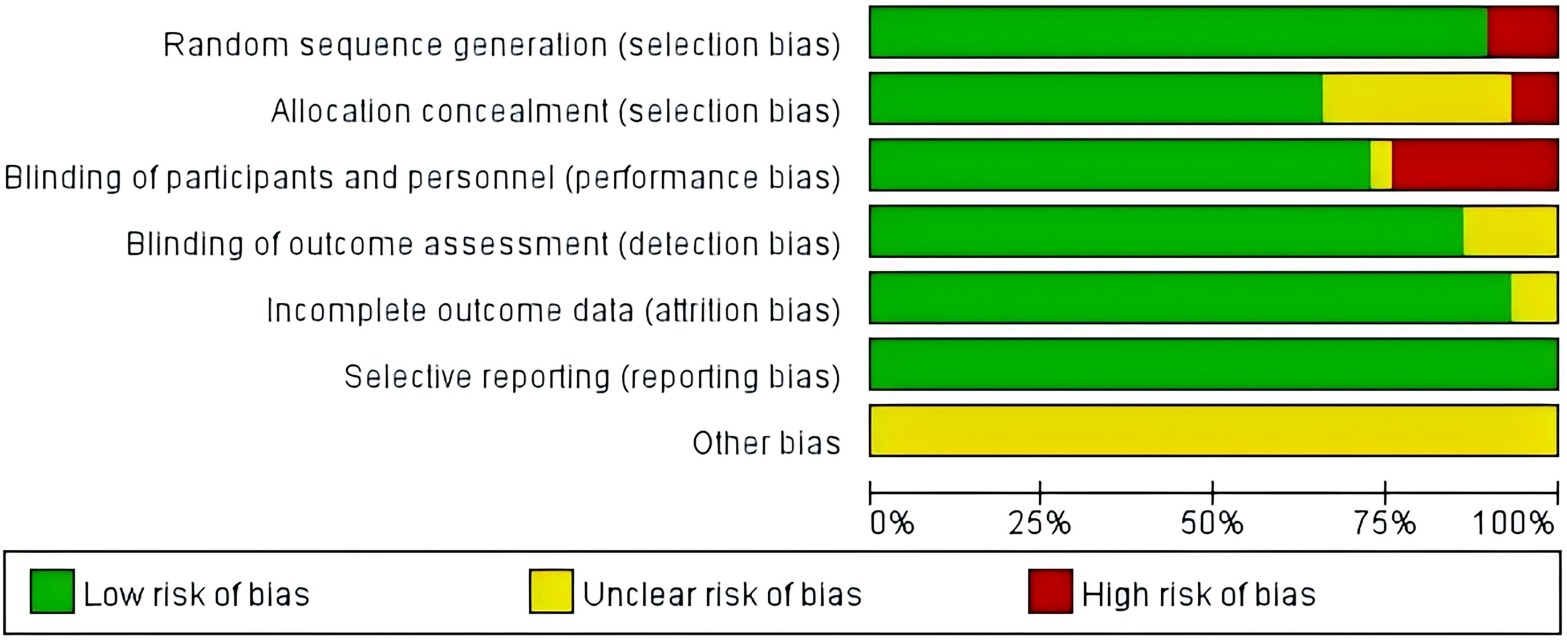
Risk of bias for inclusion in randomized controlled trials.

Twenty-nine studies were assessed using the Cochrane Risk of Bias Tool (Review Manager 5.4 tool). 26 studies reported specific methods of generating randomized sequences and were rated as ‘low risk’, while the remaining 3 studies mentioned randomization but did not describe specific methods and were rated as ‘high risk’. Two studies did not mention the implementation of allocation concealment and were rated as ‘high risk’, and eight studies mentioned blinding, but did not describe allocation concealment or blinding for outcome evaluation and were rated as ‘unclear risk’. In addition, 7 studies did not mention blinding and were rated as ‘high risk’. 29 studies reported on the expected outcome measures and were rated as ‘low risk’. 29 studies did not describe other biases in detail and were rated as ‘unclear risk’. The results of the literature quality assessment are shown in **Figure 2**.

### 3.4 Certainty of evidence

GRADEpro was used in this study to assess the certainty of evidence. **Table 2** shows that OVERALL response rate and BSFS change values are high quality evidence and other outcomes are moderate quality evidence. High heterogeneity, low methodological quality, and high risk of bias for some outcomes may have contributed to the low quality of evidence.

**Table 2:** grading of recommendations assessment, development and evaluation (CI, confidence interval; MD: mean difference; RR, risk ratio).

Quality assessment							Summary of Findings				
Participants (studies) Follow up	Risk of bias	Inconsistency	Indirectness	Imprecision	Publication bias	Overall quality of evidence	Study event rates (%)		Relative effect (95% CI)	Anticipated absolute effects	
							With WithControl Constipation			Risk with Risk difference withControl Constipation (95% CI)	
**Overall response rate** (CRITICAL OUTCOME)											
2928 (12 studies)	serious	no serious inconsistency	no serious indirectness	no serious imprecision	undetected	⊕⊕⊕⊕ HIGH due to risk of bias, large effect	255/1448 (17.6%)	552/1480 (37.3%)	OR 2.93 (2.46 to 3.5)	Study population	
										176 per 1000	209 more per 1000 (from 169 more to 252 more)
										Moderate	
										171 per 1000	206 more per 1000 (from 166 more to 248 more)
**Adverse event** (IMPORTANT OUTCOME)											
2910 (10 studies)	serious	no serious inconsistency	serious	no serious imprecision	undetected	⊕⊕⊕⊝ MODERATE due to risk of bias, indirectness, large effect	139/1447 (9.6%)	161/1463 (11%)	OR 1.19 (0.92 to 1.53)	Study population	
										96 per 1000	16 more per 1000 (from 7 fewer to 44 more)
										Moderate	
										178 per 1000	27 more per 1000 (from 12 fewer to 71 more)
**BSFS** (CRITICAL OUTCOME; Better indicated by lower values)											
3202 (16 studies)	serious	no serious inconsistency	no serious indirectness	no serious imprecision	undetected	⊕⊕⊕⊕ HIGH due to risk of bias, large effect	1612	1590	–		The mean bsfs in the intervention groups was 0.24 higher (0.22 to 0.25 higher)
**SBM** (IMPORTANT OUTCOME; Better indicated by lower values)											
3169 (14 studies)	serious	no serious inconsistency	no serious indirectness	no serious imprecision	undetected	⊕⊕⊕⊝ MODERATE due to risk of bias	1587	1582	–		The mean sbm in the intervention groups was 0.78 higher (0.76 to 0.8 higher)
**CSBM** (IMPORTANT OUTCOME; Better indicated by lower values)											
2937 (11 studies)	serious	no serious inconsistency	no serious indirectness	no serious imprecision	undetected	⊕⊕⊕⊝ MODERATE due to risk of bias	1476	1461	–		The mean csbm in the intervention groups was 0.75 higher (0.73 to 0.77 higher)
**PAC-SYM** (CRITICAL OUTCOME; Better indicated by lower values)											
543 (7 studies)	serious	no serious inconsistency	no serious indirectness	no serious imprecision	undetected	⊕⊕⊕⊝ MODERATE due to risk of bias	270	273	–		The mean pac-sym in the intervention groups was 0.3 lower (0.35 to 0.25 lower)
**PAC-QOL** (IMPORTANT OUTCOME; Better indicated by lower values)											
3058 (13 studies)	serious	no serious inconsistency	no serious indirectness	no serious imprecision	undetected	⊕⊕⊕⊝ MODERATE due to risk of bias	1514	1544	–		The mean pac-qol in the intervention groups was 0.42 lower (0.43 to 0.42 lower)

## 4 Network meta-analysis

### 4.1 Overall response rate

**Figure 3:**
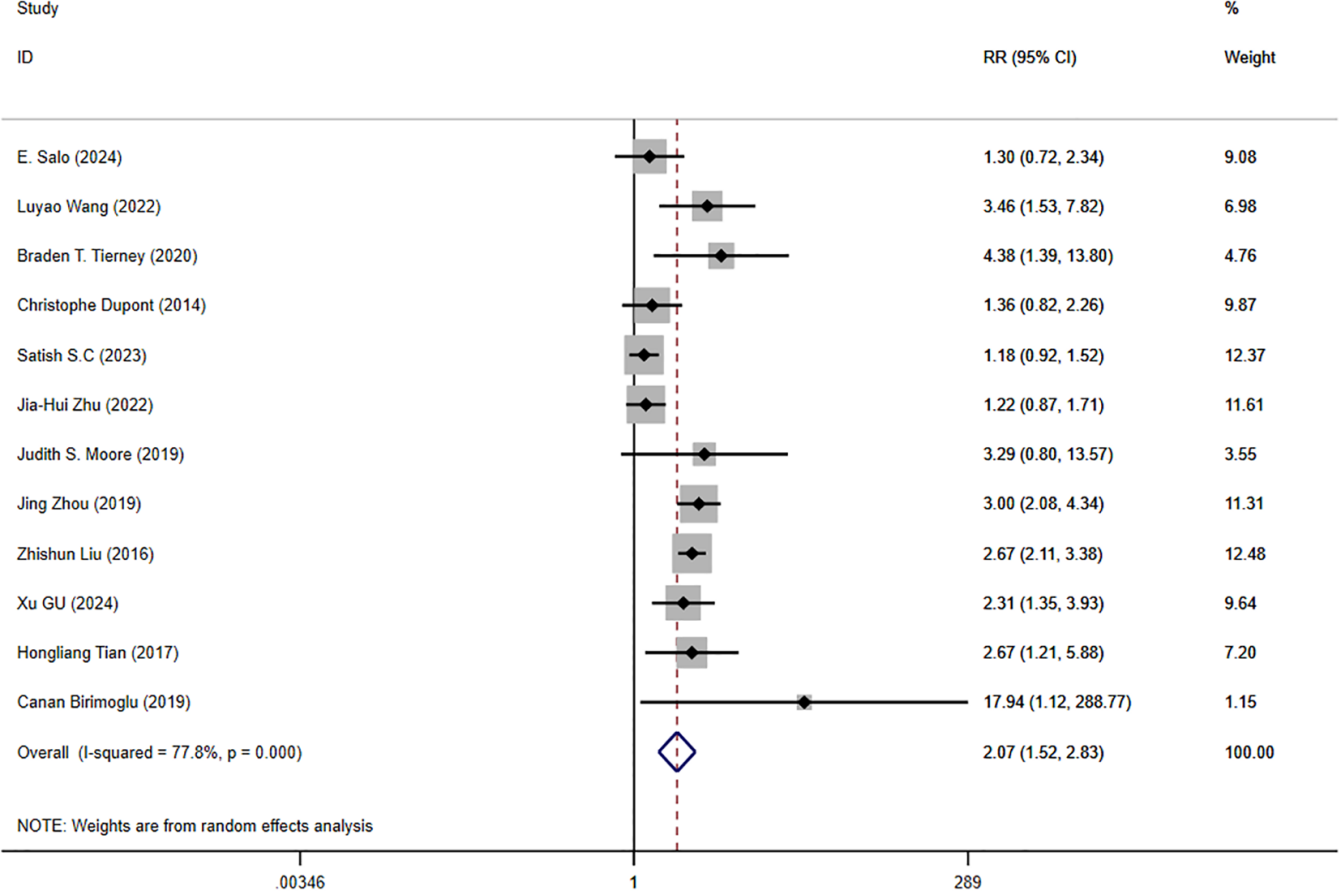
Forest plot of clinical efficacy data for trial and control group participation. RR, Risk Ratio.

There were 12 studies that reported clinical efficacy, and there was significant heterogeneity among the intervention measures (P < 0.001, I² = 77.8%). Therefore, we used a random effects model for meta-analysis. The results showed that there was a significant difference in clinical efficacy between the intervention group and the control group, with statistical significance (**Figure 3**) [RR = 2.07, 95%CI (1.52, 2.83), P < 0.001], so conducting a network meta-analysis is meaningful.

#### 4.1.1 Network evidence graph

**Figure 4:**
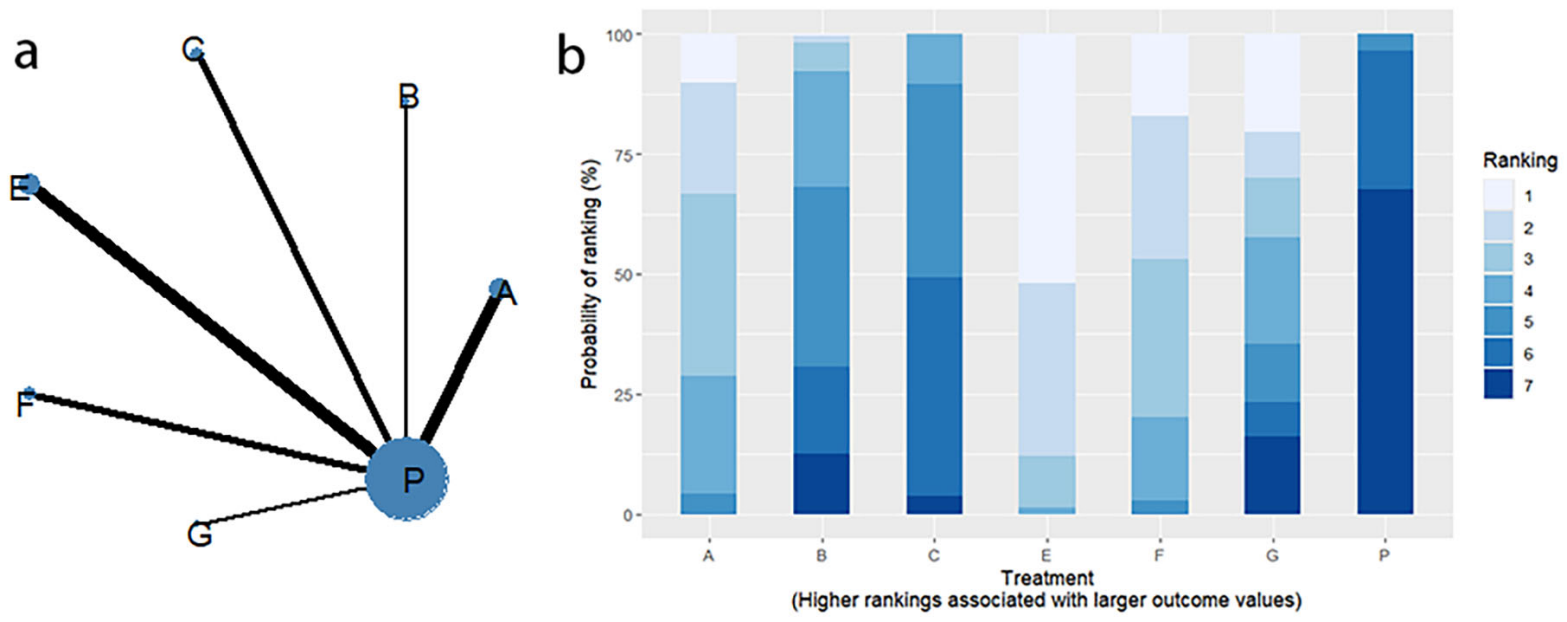
**(a)** network evidence; **(b)** rankogram figure.

**Figure 5:**
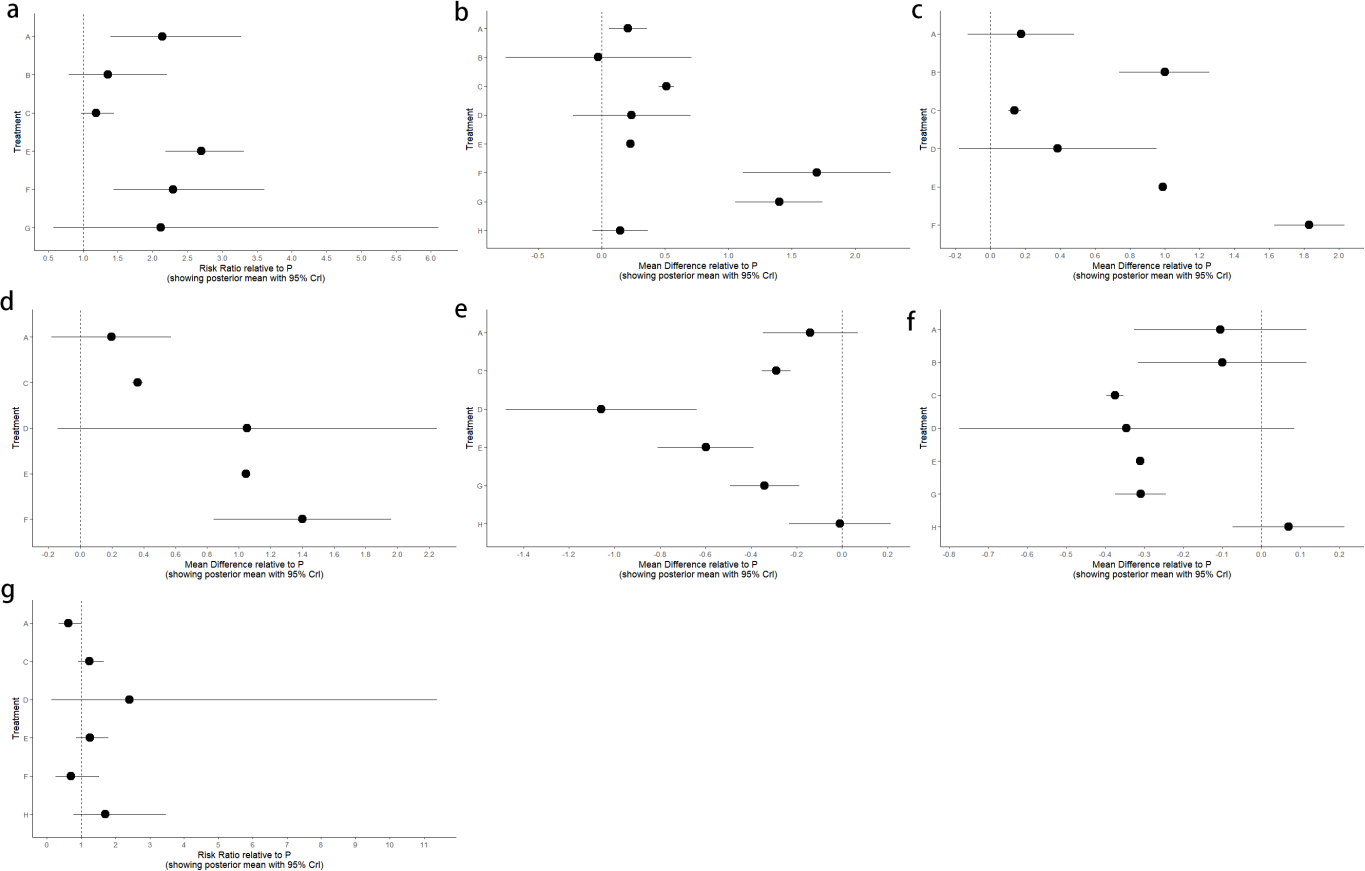
Forest plot of placebo P compared with different interventions. **(a)** Overall response rate; **(b)** BSFS change; **(c)** SBM change; **(d)** CSBM Change; **(e)** PAC-QOL Change; **(f)** PAC-SYM Change; **(g)** Adverse event.

Twelve RCTs reported clinical efficacy, including seven interventions (A: Probiotics, B: Diet, C: Vibration capsule, E: Electroacupuncture, F: FMT, G: Massage, P: Placebo), with the overall network centered around placebo treatment. The dots represent the interventions, and the lines connecting the dots represent direct comparisons between the interventions. The thickness of the lines represents the number of studies included, and the evidence network is shown in **Figure 4a**. Forest plots comparing placebo P with each intervention are shown in **Figure 5a**. It can be seen that there is no closed loop among the interventions, so no node analysis is needed.

#### 4.1.2 NMA

**Figure 6:**
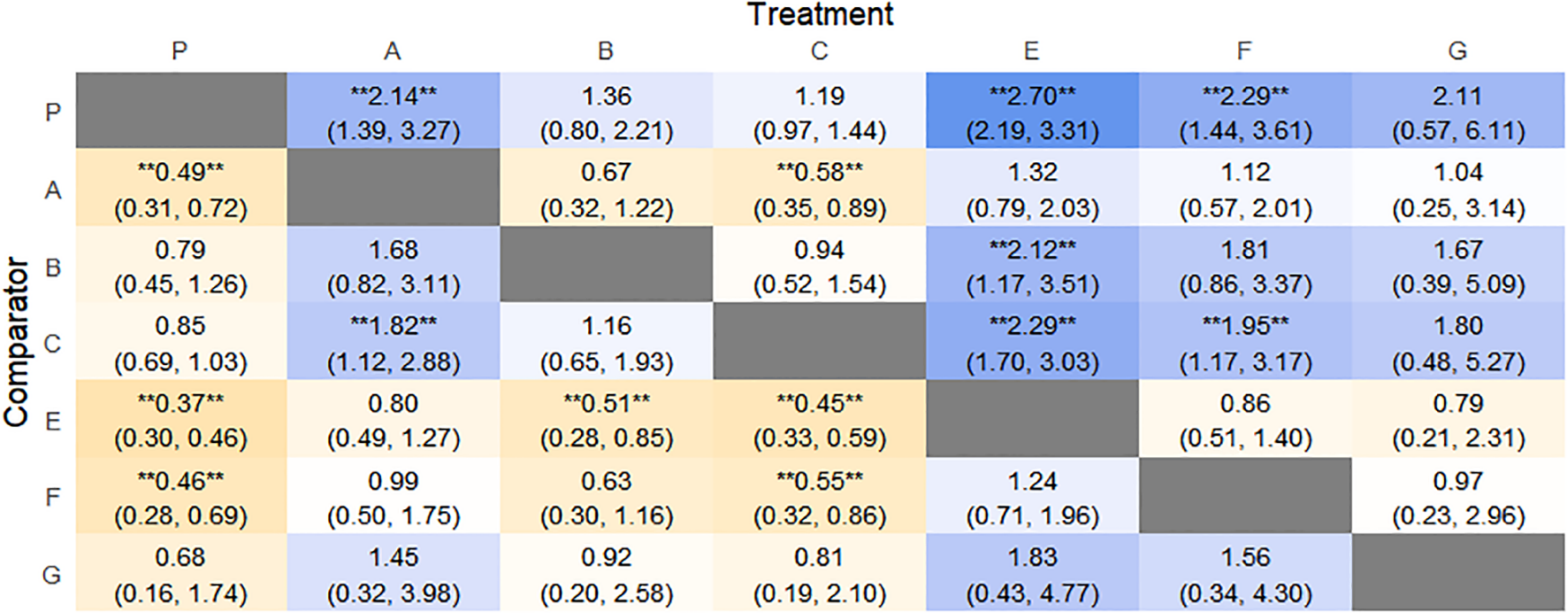
Heat map of network meta-analysis of different interventions for clinical cure rates in patients with constipation. **indicates statistical significance.

The results of network meta-analysis showed that 21 pairwise comparisons were generated, and the results of network analysis of clinical response rate were as follows (**Figure 6**).

#### 4.1.3 Efficacy ranking

**Figure 7:**
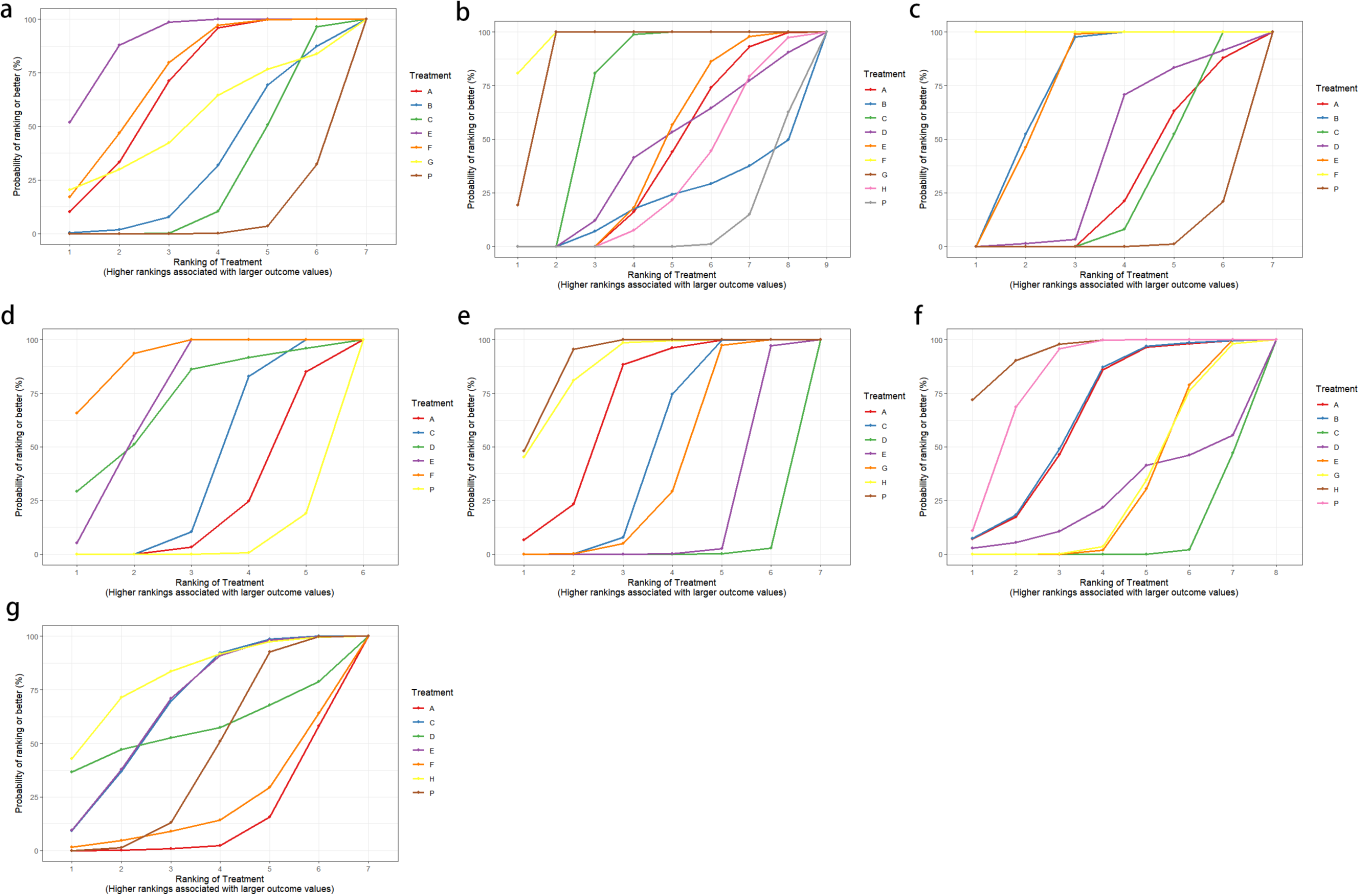
Heat map of network meta-analysis of each outcome index. **(a)** Overall response rate; **(b)** BSFS change; **(c)** SBM change; **(d)** CSBM Change; **(e)** PAC-QOL Change; **(f)** PAC-SYM Change; **(g)** Adverse event.

SUCRA probability ranking showed Electroacupuncture (84.4%) > FMT (72.4%) > Probiotics (69.6%) > Massage (63.1%) > Diet (32.1%) > Vibration capsule (24.1%) > Placebo (4%). The higher probability indicates better clinical outcomes for patients with chronic constipation, the rankogram is shown in **Figure 4b**, and the SUCRA graph is shown in (**Figure 7a**).

### 4.2 BSFS change

#### 4.2.1 Network evidence graph

**Figure 8:**
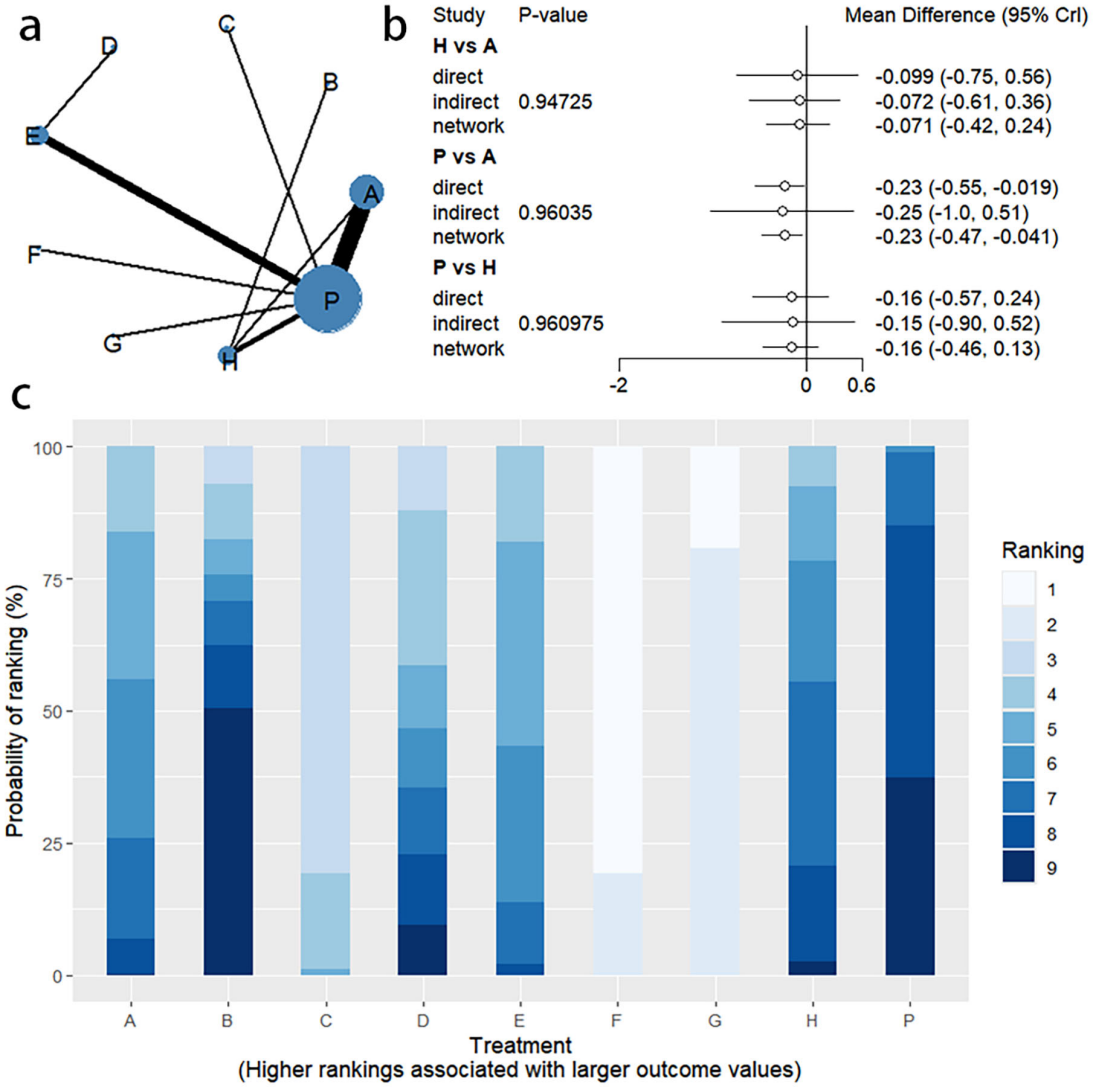
**(a)** network evidence diagram; **(b)** node analysis diagram; **(c)** rankogram figure.

Changes in BSFS were reported in 16 RCTs, including 9 interventions (A: Probiotics, B: Diet, C: Vibration capsule, D: Percutaneous electrical stimulation, E: probiotics, B: Diet, C: vibration capsule, E: percutaneous electrical stimulation). Electroacupuncture, F: FMT, G: Massage, H: Fibers, P: Placebo). The network relationship is shown in **Figure 8a**. The results of node analysis of the closed loop showed that there was no significant difference between the results of direct comparison and indirect comparison (P > 0.05), and node analysis is shown in **Figure 8b**. Forest plots comparing placebo P with each intervention are shown in **Figure 5b**.

#### 4.2.2 NMA

**Figure 9:**
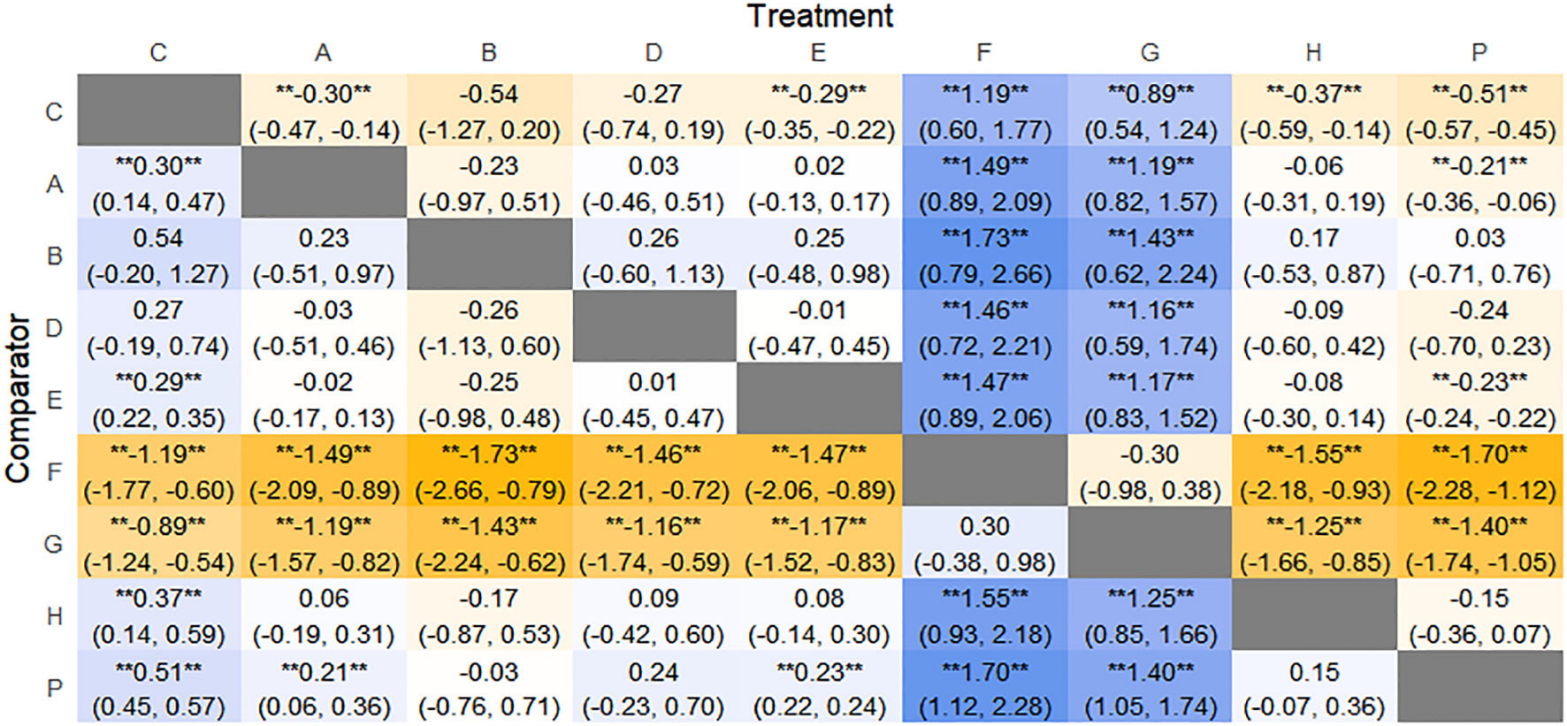
Heat map of network meta-analysis of different interventions for BSFS scores in patients with constipation. **indicates statistical significance.

The results of the reticulated Meta-analysis showed 36 two-by-two comparisons were generated, and the results of the reticulated analysis of the BSFS change values were as follows (**Figure 9**).

#### 4.2.3 Efficacy ranking

SUCRA probability ranking showed that FMT (97.5%) > Massage (89%) > Vibration capsule (72.4%) > Electroacupuncture (44.8%) > Electroacupuncture (42.3%) > Probiotics (40.9%) >Fibers (31.2%) > Diet (20.8%) > Placebo (9.8%), with a higher probability indicating a greater increase in BSFS compared to the baseline value, the rankogram is shown in **Figure 8c**, the SUCRA plot is shown in (**Figure 7b**).

### 4.3 SBM change

#### 4.3.1 Network evidence graph

**Figure 10:**
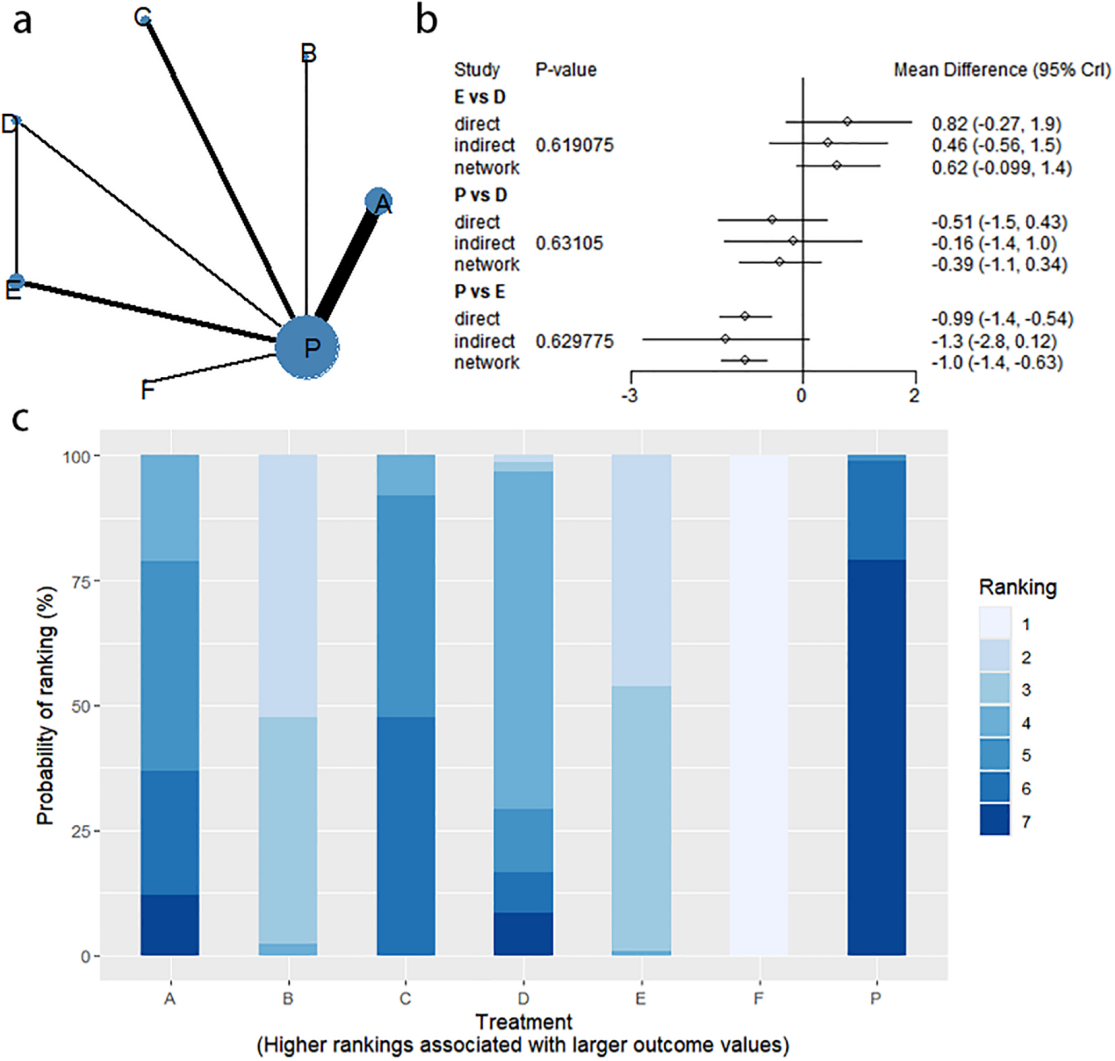
**(a)** network evidence diagram; **(b)** node analysis diagram; **(c)** rankogram figure.

Fourteen studies of RCTs reported SBM change values, including eight interventions (A: Probiotics, B: Diet, C: Vibration capsule, D: Percutaneous electrical stimulation, E: Electroacupuncture, F:FMT, P: Placebo), the network relationships are shown in **Figure 10a**, and the results of the nodal analysis of the closed loop showed that the difference between the results of the direct and indirect comparisons was not statistically significant (p > 0.05), and the nodal analysis is shown in **Figure 10b**. Forest plots comparing placebo P with each intervention are shown in **Figure 5c**.

#### 4.3.2 NMA

**Figure 11:**
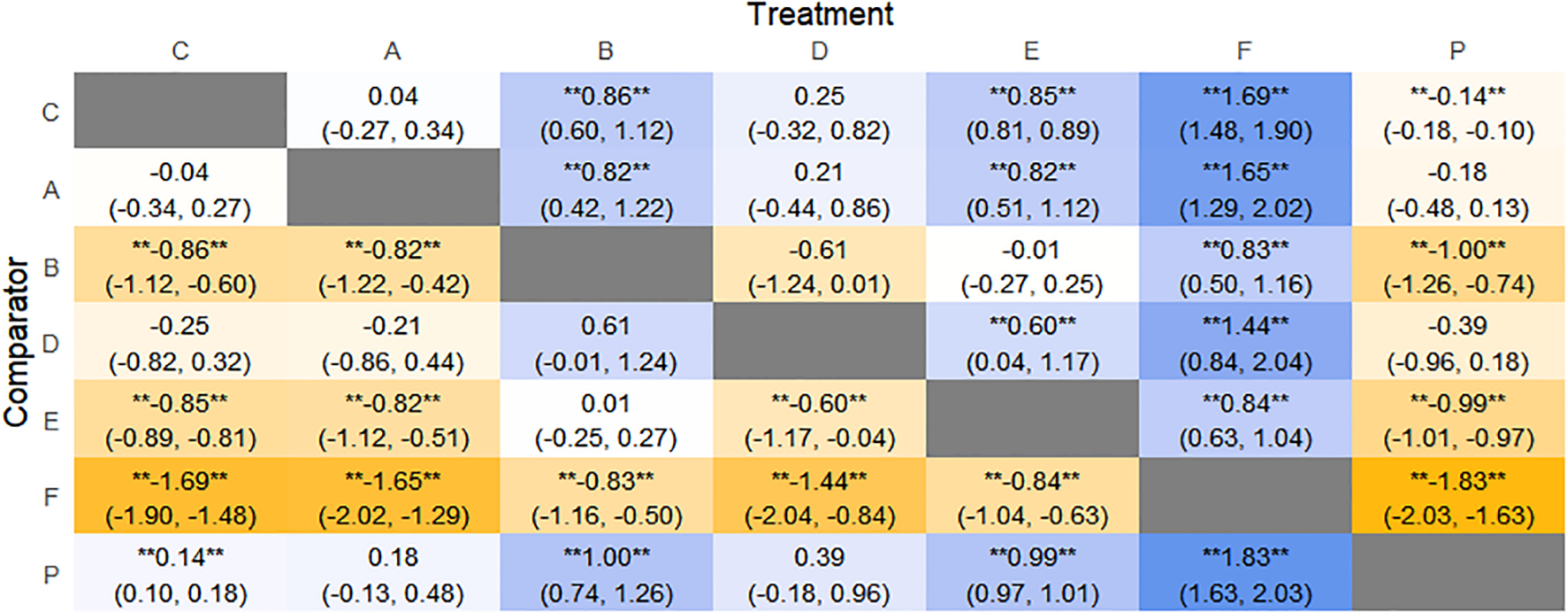
Heat map of network meta-analysis of different interventions for SBM in patients with constipation. **indicates statistical significance.

The results of network meta-analysis showed that 28 pairwise comparisons were generated, and the results of network analysis of SBM change values were as follows (**Figure 11**).

#### 4.3.3 Efficacy ranking

SUCRA probability ranking showed FMT (99.9%) > Electroacupuncture (76.3%) > Diet (73.1%) > Fibers (47.7%) > Percutaneous electrical stimulation (41.3%) > Vibration capsule (25.2%) > placebo (4%). The higher the probability, the greater the increase in SBM compared with the baseline value, the rankogram is shown in **Figure 10c**, and the SUCRA graph is shown in (**Figure 7c**).

### 4.4 CSBM change

#### 4.4.1 Network evidence graph

**Figure 12:**
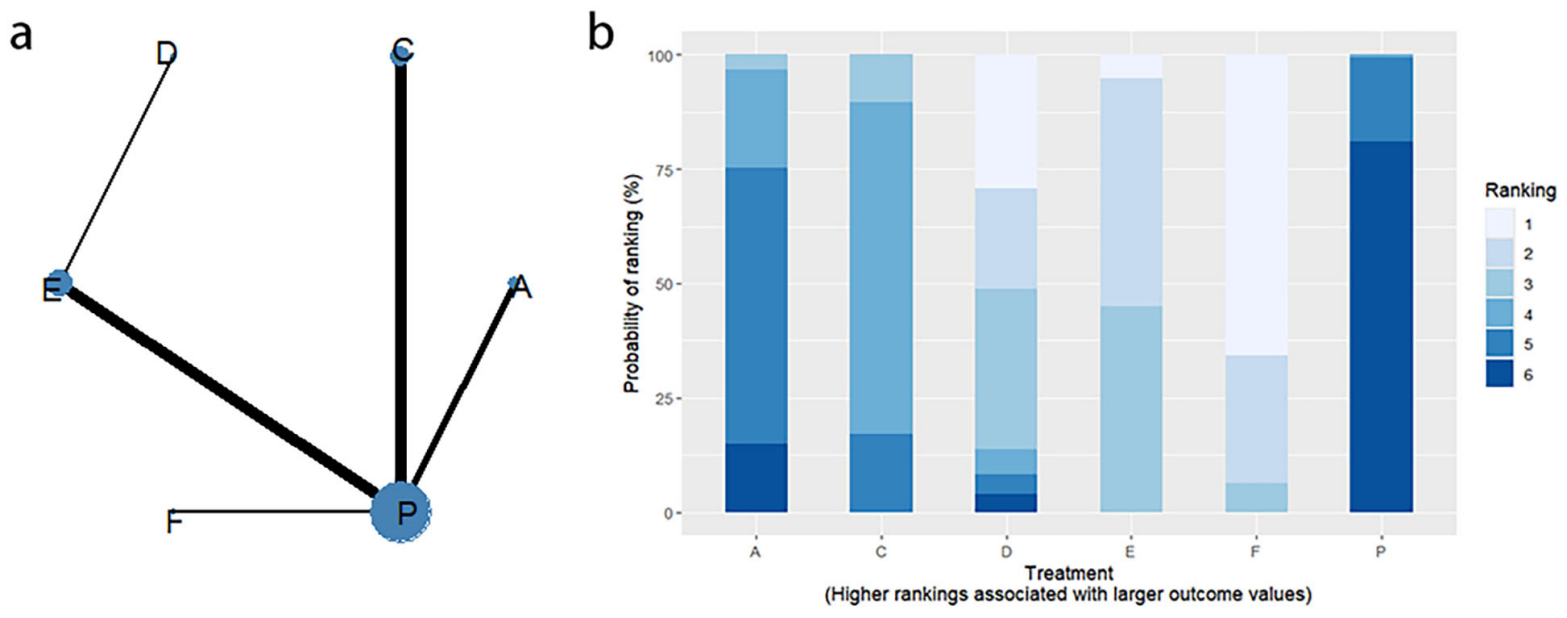
**(a)** network evidence; **(b)** rankogram figure.

CSBM change values were reported in 11 RCTs, including 6 interventions (A: Probiotics, C: Vibration capsule, D: Percutaneous electrical stimulation, E: Electroacupuncture, F: FMT, P: Placebo). The network relationship is shown in **Figure 12a**. No closed loop was formed between the various interventions, so no node analysis was needed. Forest plots comparing placebo P with each intervention are shown in **Figure 5d**.

#### 4.4.2 NMA

**Figure 13:**
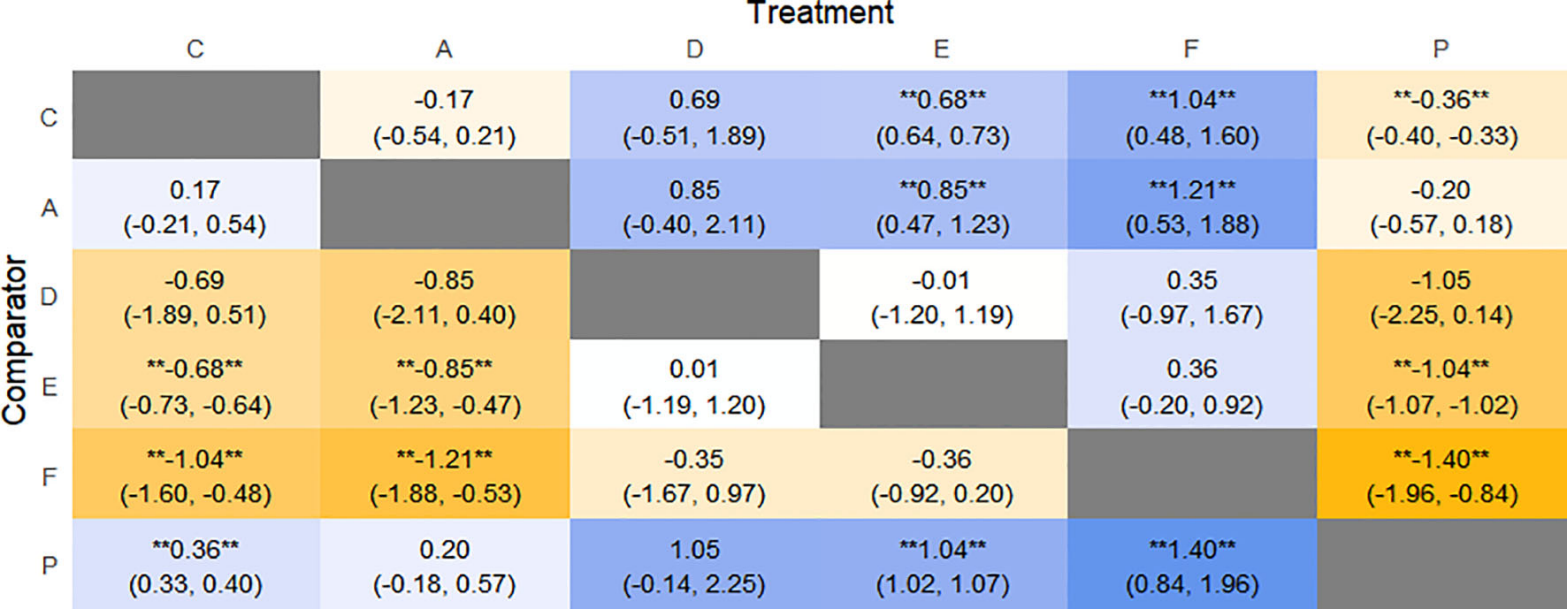
Heat map of network meta-analysis of different interventions for CSBM in patients with constipation. **indicates statistical significance.

The results of network meta-analysis showed that 15 pairwise comparisons were generated, and the results of network analysis of CSBM change values were as follows (**Figure 13**).

#### 4.4.3 Efficacy ranking

SUCRA probability ranking showed FMT (91.8%) > Electroacupuncture (72.1%) > Percutaneous electrical stimulation (70.3%) > Vibration capsule (38.7%) > Probiotics (22.7%) > placebo (4%). The higher the probability, the greater the increase in SBM compared with the baseline value, the rankogram is shown in **Figure 12b**, and the SUCRA graph is shown in (**Figure 7d**).

### 4.5 PAC-QOL change

#### 4.5.1 Network evidence graph

**Figure 14:**
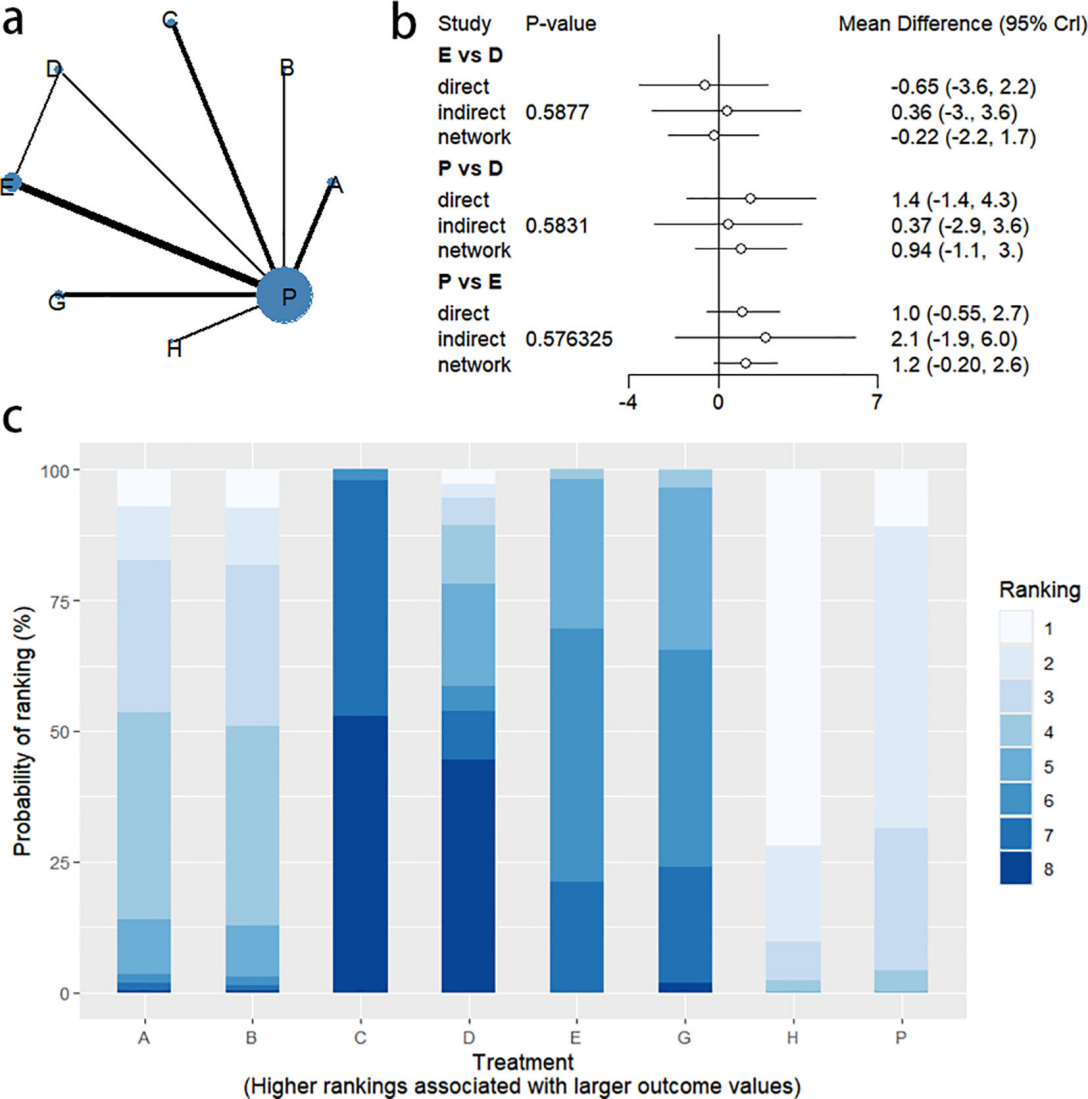
**(a)** network evidence diagram; **(b)** node analysis diagram; **(c)** rankogram figure.

Thirteen RCTs reported changes in PAC-QOL scores from baseline, including eight interventions (A: Probiotics, B: Diet, C: Vibration capsule, D: Percutaneous electrical stimulation, E: probiotics, B: Diet, C: vibration capsule, E: percutaneous electrical stimulation). Electroacupuncture, G: Massage, H: Fibers, P: Placebo). The network relationship is shown in **Figure 14a**. The results of node analysis of the closed loop showed that there was no significant difference between the results of direct comparison and indirect comparison (P > 0.05), and node analysis is shown in **Figure 14b**. Forest plots comparing placebo P with each intervention are shown in **Figure 5e**.

#### 4.5.2 NMA

**Figure 15:**
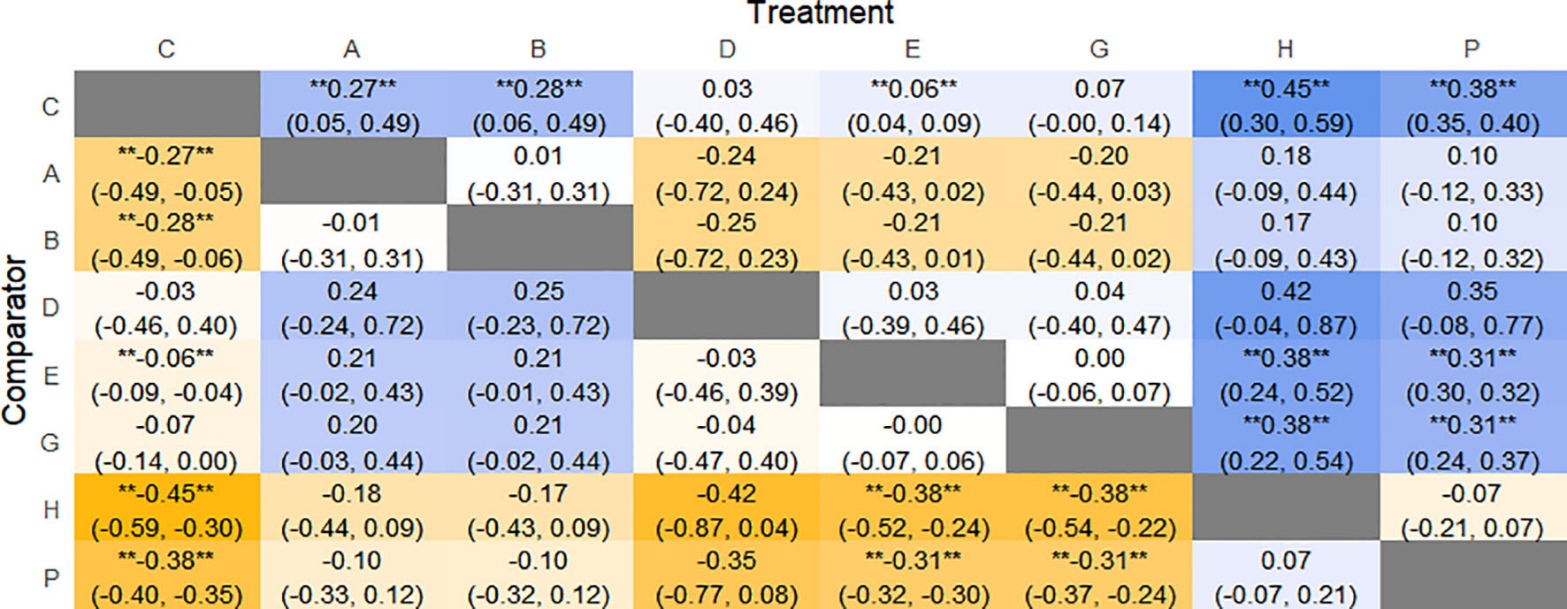
Heat map of network meta-analysis of different interventions for PAC-QOL in patients with constipation. **indicates statistical significance.

The results of the reticulated Meta-analysis showed 28 two-by-two comparisons were generated, and the results of the reticulated analysis of the value of change in PAC-QOL scores were as follows (**Figure 15**).

#### 4.5.3 Efficacy ranking

SUCRA probability ranking showed that Vibration capsule (7%) < Percutaneous electrical stimulation (26.1%) < Electroacupuncture (30.1%) < Massage (30.4%) < Probiotics (64.3%) < Diet (65.3%) < Diet (82.2%) < Fibers (94.2), the smaller the probability ranking the higher the quality of life after treatment.) <Diet (65.3%) <Diet (82.2%) <Fibers (94.2), with a smaller probability ranking indicating a higher quality of life after treatment, the rankogram is shown in **Figure 14c**, and the SUCRA graph is shown in (**Figure 7e**).

### 4.6 PAC-SYM change

**Figure 16:**
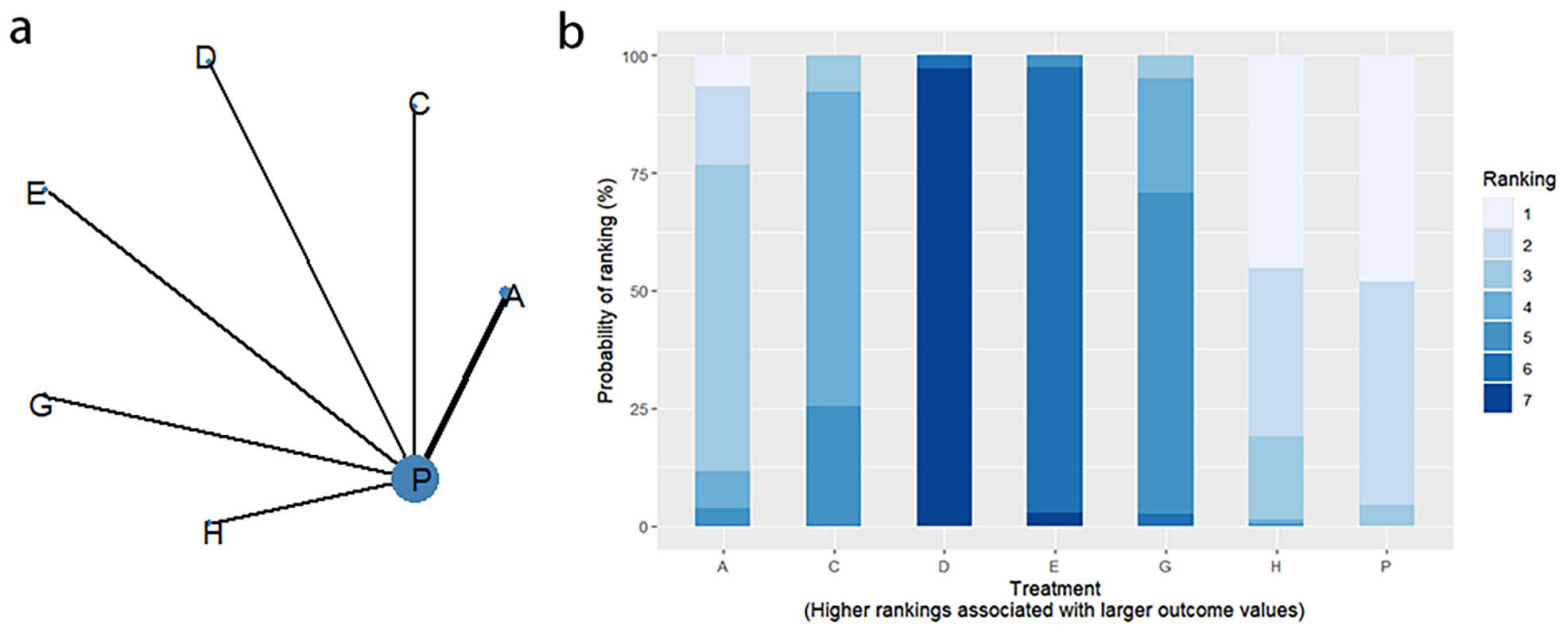
**(a)** network evidence; **(b)** rankogram figure.

Seven studies of RCTs reported values of change from baseline in PAC-SYM, including seven interventions (A: Probiotics, C: Vibration capsule, D: Percutaneous electrical stimulation, E: Electroacupuncture, G:Massage, H: Fibers, P: Placebo), and the network relationships are shown in **Figure 16a**; no closed loop was formed between the interventions and no nodal analysis was required. Forest plots comparing placebo P with each intervention are shown in **Figure 5f**.

#### 4.6.1 NMA

**Figure 17:**
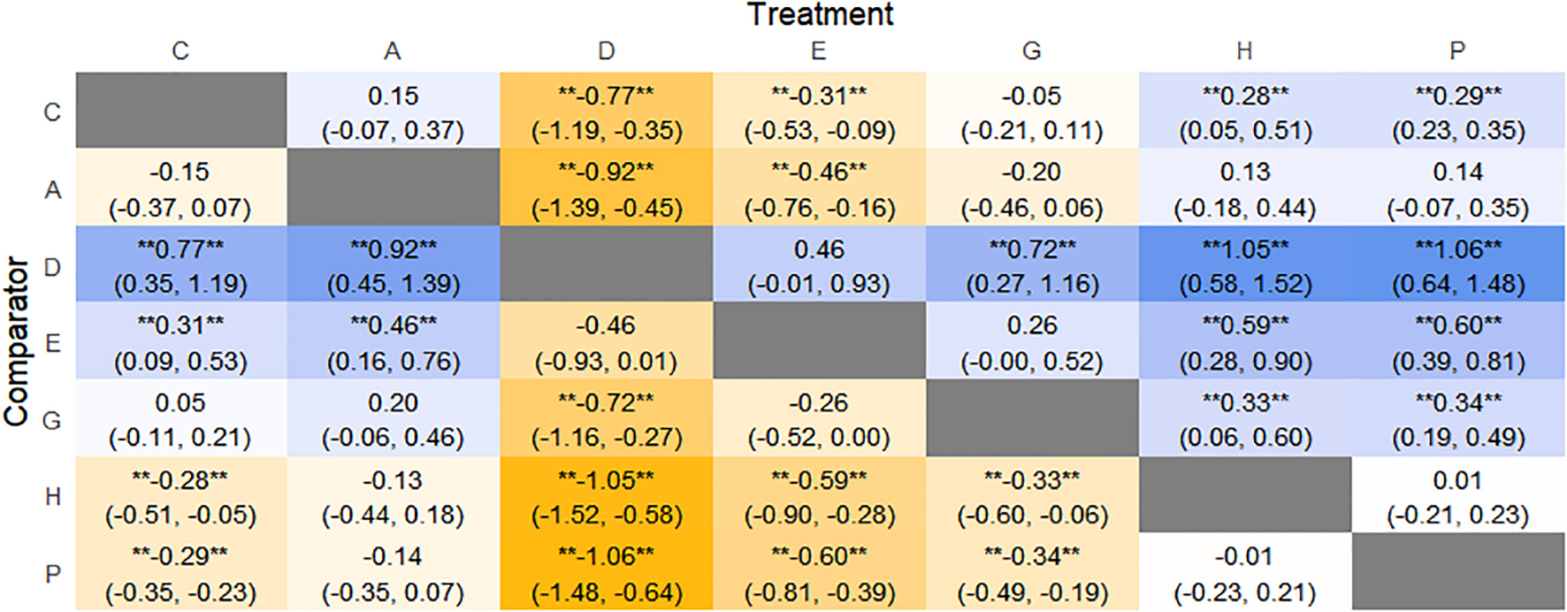
Heat map of network meta-analysis of different interventions for PAC-SYM in patients with constipation. **indicates statistical significance.

The results of network meta-analysis showed that 21 pairwise comparisons were generated, and the results of network analysis of PAC-SYM and baseline changes were as follows (**Figure 17**).

#### 4.6.2 Efficacy ranking

SUCRA probability ranking showed that Percutaneous electrical stimulation (0.4%) < Electroacupuncture (16.7%) < Massage (38.6%) < Vibration capsule (47.1%) < Probiotics (69%) < Fibers (87.3%) < Placebo (90.6%). The smaller the probability, the larger the change value of PAC-SYM, the rankogram is shown in **Figure 16b**, and the SUCRA graph is shown in (**Figure 7f**).

### 4.7 Adverse event

**Figure 18:**
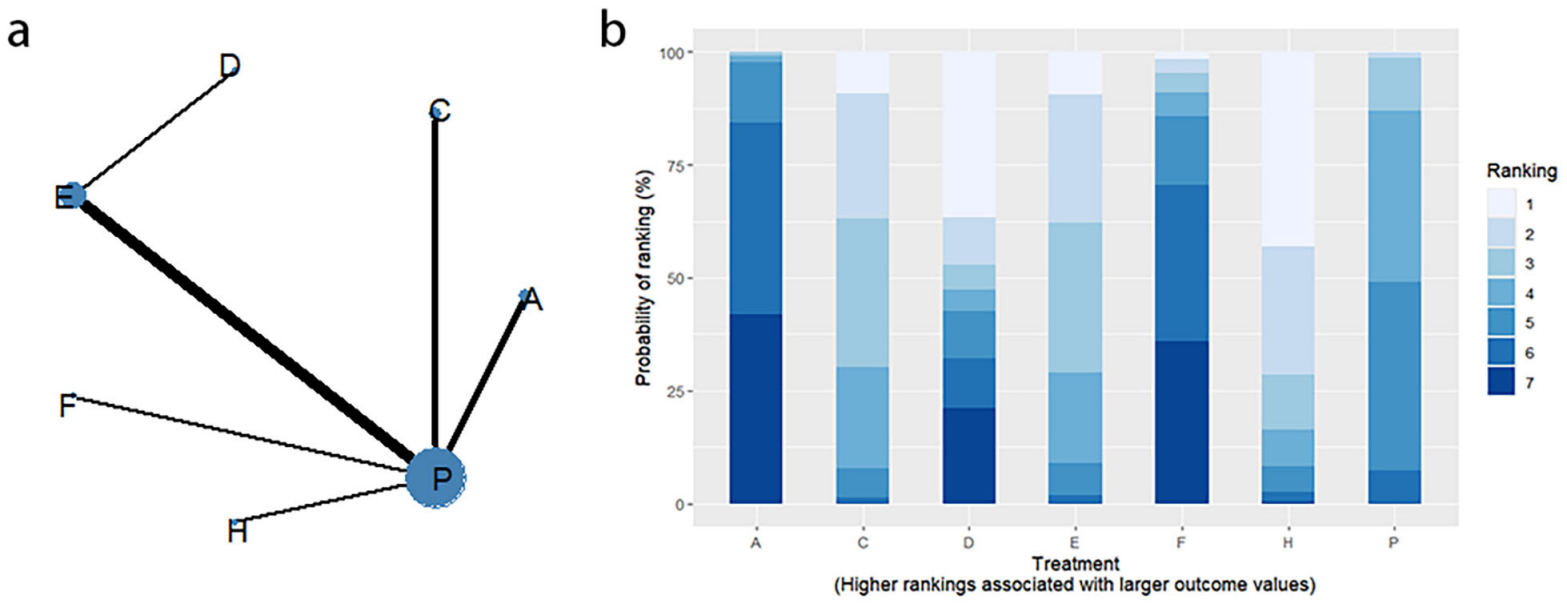
**(a)** network evidence; **(b)** rankogram figure.

Adverse events were reported in 10 RCTs, including 7 interventions (A: Probiotics, C: Vibration capsule, D: Percutaneous electrical stimulation, E: Electroacupuncture, F: FMT, H: Fibers, P: Placebo). The network relationship is shown in **Figure 18a**. No closed loop was formed between the various interventions, and node analysis was not needed. Forest plots comparing placebo P with each intervention are shown in **Figure 5g**.

#### 4.7.1 NMA

**Figure 19:**
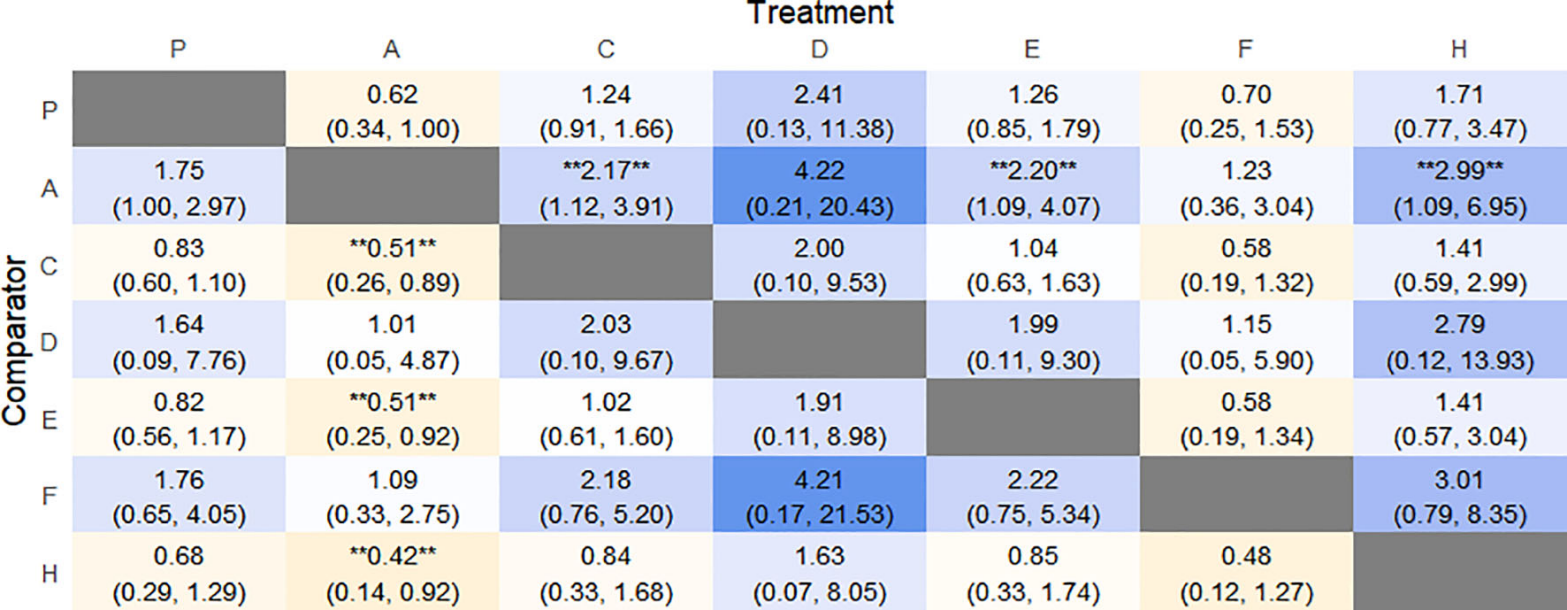
Heat map of network meta-analysis of different interventions for adverse event in patients with constipation. **indicates statistical significance.

The results of network meta-analysis showed that 21 pairwise comparisons were generated, and the results of network analysis of adverse events were as follows (**Figure 19**).

#### 4.7.2 Efficacy ranking

The probability of SUCRA was Fibers (86.4%) > Electroacupuncture (73.2%) > Vibration capsule (69.8%) > Placebo (44.1%) > Percutaneous electrical stimulation (32%) > FMT (26.8%) > Probiotics (17.5), the smaller the probability, the less adverse events, the rankogram is shown in **Figure 18b**, and the SUCRA graph is shown in (**Figure 7g**).

### 4.8 Forest plot of placebo P compared with each intervention

Forest plot of the comparison between placebo P and each intervention in the outcome measures Overall response rate, Adverse event, BSFS score, SBM change, CSBM change, PAC-SYM score, and PAC-QOL score (**Figure 5**).

### 4.9 Heat map of network meta-analysis

SUCRA plots of each intervention in the outcome measures total effective rate, adverse events, BSFS score, SBM change, CSBM change, PAC-SYM score, and PAC-QOL score (**Figure 7**).

## 5 Subgroup analyses of response rates

We stratified the severity of constipation according to the patient’s baseline SBM value and performed subgroup analysis to show the effect of baseline constipation severity on treatment effect (**Table 3**). However, due to the lack of literature on some interventions, we can only conclude that for probiotic interventions, baseline SBM ≥3 May have a lower response rate. In addition, subgroup analysis was conducted according to patients’ age, and the results showed that the effect of probiotics and FMT treatment was better in patients aged ≥45 years. The median mean age and median SBM mean of the included studies were used for age and baseline SBM cutoff values.

**Table 3:** The results were analyzed by two different methods in age and baseline SBM of patients with constipation treated by intervention measures.

As compared with placebo	Network Meta-analysis	Subgroup analysis			
		Age<45	Age≥45	SBM<3	SBM≥3
Probiotics	2.3(1.5,3.8)	1.9(1.1,3.4)	3.6(1.7,9.3)	2.3(1.5,3.8)	1.9(1.1,3.4)
Diet	1.4(0.83,2.3)	1.4(0.83,2.3)	NA	1.4(0.83,2.3)	1.4(0.82,2.3)
Vibration capsule	1.2(0.98,1.5)	1.2(0.87,1.7)	1.2(0.93,1.5)	1.2(0.98,1.5)	NA
Electroacupuncture	2.8(2.2,3.4)	NA	2.8(2.2,3.4)	2.8(2.2,3.4)	NA
FMT	2.4(1.5,4.0)	2.3(1.4,4.2)	2.9(1.1,10.0)	2.4(1.5,4.0)	NA
Massage	2.2(0.68,9.7)	NA	2.2(0.68,9.7)	2.2(0.68,9.7)	NA

## 6 Discussion

The global incidence of FC has been rising, attributed to shifts in dietary habits and lifestyle changes. The incidence of FC has been increasing globally due to changes in dietary habits and lifestyles. In addition, with the innovation of constipation diagnostic technology, high-resolution anorectal manometry, wireless motion capsule colonic transmission testing and artificial intelligence-assisted symptom analysis have led to a significant increase in clinical recognition of constipation. Compared with the traditional symptom-based Rome III criteria, which may be difficult to fully diagnose subclinical or mixed constipation, the updated Rome IV criteria have allowed more occult patients to be accurately identified through the integration of biomarkers such as anorectal coordination disorders and quantitative assessment tools such as BSFS and PAC-SYM. In China, the overall prevalence of FC is reported to be 10.9%, with a higher prevalence of 11.5% observed in women (Aziz et al., 2020).Current treatment options for FC primarily include osmotic and stimulant laxatives, stool softeners, cholinesterase inhibitors, and pro-secretory agents such as lactulose, mosapride, magnesium sulfate, polyethylene glycol, and bisacodyl. However, the long-term or inappropriate use of these medications can lead to adverse effects, including exacerbation of constipation symptoms, drug dependence, and vitamin absorption disorders, which can contribute to recurrent symptoms and poorer patient outcomes (Bharucha and Lacy, 2020; Serra et al., 2020b).As modern medical research progresses, the understanding of FC has transitioned from being perceived solely as a digestive disorder within the biomedical model to being recognized as a psychosomatic condition within the biopsychosocial model. This paradigm shift underscores the importance of brain-gut interactions in the pathogenesis of FC (Agirman et al., 2021). Recent advancements in microbiological research and the development of various histological techniques have underscored the crucial role of intestinal microecology in FC. The imbalance of intestinal flora will not only damage the intestinal environment, but also reduce the production of metabolites such as short-chain fatty acids butyric acid and propionic acid. The imbalance of microbiota - gut - brain axis can also down-regulate the secretion of neurotransmitters such as 5-HT and disrupt the signal transduction of the intestinal nervous system (Erhardt et al., 2023). Additionally, non-pharmacological treatment approaches targeting intestinal dysmotility, the brain-gut axis, and the gut microbiota have been developed. However, the therapeutic effects of many of these modalities remain unclear. To evaluate the clinical efficacy and safety of various non-pharmacological interventions for functional constipation, this study employs a network meta-analysis to compare the effectiveness and incidence of adverse reactions associated with nine different treatments. The findings aim to provide valuable insights to inform clinical decision-making.

We conducted a meta-analysis of 29 RCTs involving a total of 4,389 patients with FC. After data extraction, we identified eight interventions: Probiotics, Diet, Vibration Capsule, Percutaneous Electrical Stimulation, Electroacupuncture, FMT, Fiber, and Massage. Our meta-analysis revealed that all eight interventions significantly alleviated constipation symptoms compared to placebo, with a RR of 2.07 and a 95% CI of (1.52, 2.83), P < 0.001. Furthermore, the results of the network meta-analysis indicated that Electroacupuncture ranked highest in terms of clinical effectiveness. In terms of changes in stool frequency and consistency, FMT was the most effective treatment. Additionally, FMT also ranked first for changes in BSFS scores post-treatment, while Vibration ranked highest for changes in PAC-QOL scores after treatment. The pathogenesis of FC may be attributed to various factors, including a decrease in the number and amplitude of peristaltic waves in the gastrointestinal tract and insufficient transmission power of the colon (Yu et al., 2024). In 2015, Ron et al. introduced vibrating capsules as a safe and effective treatment for chronic constipation. This approach is based on the principle of stimulating peristaltic waves through mechanical impact on the intestinal wall, thereby promoting bowel movements (Ron et al., 2015). However, due to its limited vibration mode and the absence of ex vivo regulation and monitoring, this treatment has not been widely adopted in clinical practice. In 2022, Wang et al. enhanced the design of the vibrating capsule by incorporating features such as *in vivo* and ex vivo communication and a work display. They also introduced a silent period of 6-8 hours during which the capsule relies on gravity and the natural emptying mechanism of the gastrointestinal tract as it traverses the stomach and small intestine. This design minimizes swallowing discomfort and reduces energy consumption. The study results indicated that the vibrating capsule effectively alleviates constipation symptoms and improves quality of life. Furthermore, in the normal population, the multidimensional vibrating capsule was found to increase the frequency of CSBM (Wang et al., 2022). Our findings align with previous studies indicating that vibration capsules are more effective in enhancing patients’ PAC-QOL scores; however, they did not show a significant advantage in alleviating constipation symptoms. Given that vibration capsules are still in the development phase, further research is essential, particularly large-scale, high-quality randomized controlled trials. Acupuncture therapy, a fundamental aspect of complementary medicine, has gained considerable recognition both domestically and internationally for its efficacy in treating FC. Previous studies have demonstrated that electroacupuncture significantly upregulates the expression of colonic tyrosine kinase receptor (c-kit) and stem cell factor (SCF) in FC rats, increases the number of interstitial cells of Cajal (ICC), enhances their structure and function, regulates gastrointestinal motility, and induces a stimulating effect in the distal colon (Yao et al., 2022; Kuang et al., 2022). The brain-gut axis refers to the bidirectional communication pathway through which the brain interacts with the neurological, endocrine, and immune systems of the gastrointestinal tract. This interaction influences the sensory, secretory, and motor functions of the gastrointestinal (GI) tract in response to external stimuli and abnormal psychosocial factors (Stasi et al., 2012). Normal colonic dynamics depend on the balanced function of the smooth muscle, the enteric nervous system (ENS), and the brain-gut axis. Disruptions in the brain-gut axis can impair the intermuscular plexus and enteric ganglion cells, leading to pathological responses such as delayed gastric emptying and slowed intestinal peristalsis. These disruptions represent a key mechanism in the pathogenesis of FC (Song et al., 2023). Studies have demonstrated that in patients with FC, the expression of excitatory hormones such as gastrin (GAS), substance P (SP), and serotonin (5-HT) is diminished, while the expression of inhibitory hormones like somatostatin (SS), vasoactive intestinal peptide (VIP), and nitric oxide (NO) is increased in the colon (Yi et al., 2023). Acupuncture can positively influence the brain-gut axis through various mechanisms, including the regulation of nervous system function, modulation of brain-gut peptide levels, enhancement of the diversity and abundance of intestinal microbiota, and improvement of rectal hypoallergenicity (RH). These effects promote beneficial brain-gut interactions, which subsequently enhance gastrointestinal motility, restore intestinal microecology, and alleviate mood disturbances, ultimately aiding in the intervention of FC progression (Li et al., 2022). Wang et al. (2021) concluded that the application of all three types of acupuncture—water acupuncture, white acupuncture, and acupoint burrowing—could enhance intestinal peristalsis and alleviate constipation in rats. This treatment significantly elevated the expression levels of SP, type 3 and type 4 receptors for 5-HT, and SERT mRNA, while concurrently reducing the content of VIP. Furthermore, acupuncture was found to up-regulate the abundance and diversity of intestinal flora, increasing the prevalence of beneficial bacteria such as Staphylococcaceae and Lactobacillaceae, while decreasing the abundance of potentially pathogenic bacteria, including Saccharomyces cerevisiae and Escherichia coli. Additionally, acupuncture regulated the content of short-chain fatty acids (SCFAs), which subsequently alleviated the symptoms of FC (Xu et al., 2021). A meta-analysis conducted by Jun-peng demonstrated that acupuncture may be a safe and effective treatment for FC, showing superiority over medication in enhancing bowel frequency, stool formation, and overall quality of life (Yao et al., 2022). This finding aligns with our study, which similarly indicated that acupuncture is more clinically effective in treating patients with FC. In contrast, transcutaneous electrical stimulation (TENS), which employs surface electrodes to deliver current at specific points near or overlapping with peripheral nerves, has not demonstrated superior efficacy in any aspect of treatment.

Disrupted intestinal microbiota is a key factor in the pathogenesis of functional constipation. Compared to healthy individuals, patients with constipation exhibit significant differences in intestinal microorganisms at the phylum, genus, and species levels, as well as in the metabolic pathways of the microbiota. Notably, there is marked enrichment in pathways related to fatty acid synthesis and degradation, butyrate metabolism, and methane metabolism, suggesting that alterations in microbiota composition and its derived metabolites may contribute to constipation (Tian et al., 2022), Potential mechanisms include: (1) specific microbial strains participating in bile acid (BA) metabolism through enzymatic reactions. Bile acids can activate intestinal chromaffin cells, the farnesoid X receptor (FXR), and the G-protein-coupled bile acid receptor (TGR5) in endogenous primary afferent neurons, stimulating the release of serotonin (5-HT) and calcitonin gene-related peptides, thereby initiating the intestinal peristalsis reflex (Fan et al., 2022); (2) Elevated levels of SCFAs, produced through microbial fermentation, promote colonic peristalsis, increase defecation frequency, and stimulate the secretion of gastrointestinal hormones such as peptide YY (PYY) and glucagon-like peptide-1 (GLP-1) from the ileocecal terminal to regulate gastrointestinal motility. Additionally, SCFAs enhance serotonin(5-HT) synthesis by upregulating the expression of tryptophan hydroxylase-1(TPH1) (Lund et al., 2018); (3) The gut microbiota regulates 5-HT levels through multiple mechanisms, influencing colonic peristalsis and contributing to FC. (4) Methane production by the gut microbiota has been shown to reduce defecation frequency. The predominant methanogenic bacterium in the human gut, Methanobrevibacter smithii, has been found to be overrepresented in the intestines of constipated patients with elevated methane levels (Koch et al., 2017). Microecological agents, including probiotics, prebiotics, synbiotics, and fecal transplants, aim to alleviate symptoms of constipation by regulating the intestinal microbiota. Probiotics, the most commonly utilized among these agents, can enhance intestinal barrier function by modulating immune responses and preventing pathogen colonization. However, their therapeutic effects are inconsistent, with contradictory findings reported in the literature. Two randomized controlled trials investigating constipation revealed that a probiotic complex containing Lactobacillus acidophilus NCFM, Lacticaseibacillus paracasei Lpc-37, and Bifidobacterium animalis subsp. lactis HN019 did not have a significant impact on clinical indicators associated with constipation (Airaksinen et al., 2019; Ibarra et al., 2018). Our analysis corroborated these findings, indicating that the probiotic intervention exhibited weaker effects across all metrics, with only a limited number of outcomes demonstrating superiority over the placebo. This may be attributed to the fact that traditional probiotics, such as Lactobacillus and Bifidobacterium, do not play a significant role in regulating gut motility. Additionally, factors such as probiotic species, combinations, concentrations, and the duration and frequency of treatments remain unstandardized. Nevertheless, probiotic interventions are generally safe and exhibit a lower incidence of adverse events compared to other treatments. Although fiber can be fermented by gut microbiota into SCFAs, which subsequently release serotonin (5-HT) to promote intestinal motility, its clinical effectiveness is limited. Furthermore, prolonged high-fiber intake may elevate the risk of colorectal cancer (Yang et al., 2024b). FMT involves extracting feces from healthy donors, which are rich in beneficial bacteria, and then infusing the treated feces back into the patient’s body via colonoscopy, gastroscopy, or alternatively, by capsule or enema. This procedure aims to restore or improve the patient’s intestinal microbiota. The effects of FMT may vary depending on the method of administration. Chen et al. (2020) conducted FMT on 3,923 patients and found differences in the effectiveness over a 5-year follow-up, depending on the route of transplantation. However, consistent with our findings, FMT generally demonstrated a significant clinical effect, including increased bowel movement frequency and improved stool consistency. Zhang et al. (2021) retrospectively analyzed 1,000 patients with constipation admitted to the hospital for FMT treatment from 2017-2020, with a clinical effectiveness rate of 67%. Tian et al. (2020) analyzed changes in the fecal microbiota of patients with constipation, and found that there was a higher abundance of Bacillus-like organisms and Enterobacteriaceae prior to FMT, and an increased abundance of Prevotella and Acidinomycin, which apparently accelerates colonic motility. There remains a risk of recurrence following FMT, which may be attributed to variations in transplantation routes and the inability of gut bacteria to permanently colonize the host’s gut. In such cases, re-administration every 3 to 6 months may be necessary to maintain therapeutic efficacy. Given the diversity of the intestinal microbiota, the individual variability of bacterial strains, and the complex interactions between them, several challenges remain in achieving stable colonization of intestinal microorganisms. Considering that pharmacological interventions for functional constipation are associated with low safety, frequent adverse reactions, suboptimal therapeutic effects, and a high risk of patient dependence, non-pharmacological methods not only provide short-term symptom relief but also maintain intestinal health and avoid long-term risks such as laxative dependence or electrolyte imbalance. These approaches achieve this by adjusting lifestyle and physiological habits and directly targeting causative factors of constipation, including low-fiber diets, intestinal flora disorders, sedentarism, and impaired defecation reflexes. Furthermore, non-pharmacological interventions for functional constipation demonstrate advantages in patient acceptance, compliance, reduced side effects, and broader applicability. In conclusion, non-pharmacological treatments are not only economically feasible but also capable of alleviating functional constipation through multidimensional, individualized, and comprehensive interventions that reshape intestinal health ecology at its root.

This is the first network meta-analysis of non-pharmacological modalities for the treatment of FC, and the included literature is relatively comprehensive, excluding older studies. As a result, the findings of this article are fairly reliable. However, there are several limitations: (1) the included studies do not standardize the severity of constipation among patients, which could introduce a potential risk of bias; (2) some interventions are represented by a relatively small number of studies, which may result in variability in the results;(3)Limited by the study design and relatively centralized age distribution, no adjustment for stratification of patients according to baseline severity of functional constipation, and insufficient literature on some interventions, although we performed subgroup analyses by age and baseline SBM, the results were not very significant. We hope that future analyses will incorporate more high-quality, multicenter randomized controlled trials to address these limitations.

## 7 Conclusion

Based on the 29 included randomized controlled trials, this study is the first to systematically compare the efficacy and safety of non-pharmacological interventions such as dietary fiber supplementation, probiotics, FMT, vibration capsules, abdominal massage, and acupuncture through network meta-analysis. We found that FMT had better efficacy and higher safety for BSFS score, SBM and CSBM. In addition, acupuncture has shown good clinical efficacy. We hypothesized that the combination of FMT and acupuncture for functional constipation may be an effective and safe treatment option, but further high-quality clinical studies are needed to confirm this.

## Data Availability

The original contributions presented in the study are included in the article/supplementary material. Further inquiries can be directed to the corresponding authors.
