# Correction: Clinical efficacy of non-pharmacological treatment of functional constipation: a systematic review and network meta-analysis

**DOI:** 10.3389/fcimb.2025.1680092

**Published:** 2025-08-25

**Authors:** Shufa Tan, Chengtao Peng, Xin Lin, Chuanyue Peng, Yunyi Yang, Shuang Liu, Ling Huang, Yuhong Bian, Yuwei Li, Chen Xu

**Affiliations:** ^1^ School of Intergrative Medicine, Tianjin University of Traditional Chinese Medicine, Tianjin, China; ^2^ The First Affiliated Hospital, Jiangxi Medical College, Nanchang University, Nanchang, China; ^3^ Department of Anorectal, Tianjin Union Medical Center The First Affiliated Hospital of Nankai University, Tianjin, China; ^4^ Shanghai University of Traditional Chinese Medicine, Shanghai, China; ^5^ College of Chemistry, Nankai University, Tianjin, China

**Keywords:** network meta-analysis, functional constipation, gut microbiota, chemical drugs, fecal microbiota transplantation, clinical efficacy

Author “Shufa Tan” was erroneously omitted as equal contributing author. The correct list of authors should be “Shufa Tan^1†^”, to reflect that “Shufa Tan^1†^, Chengtao Peng^2†^, Xin Lin^3†^” all made equal contributions to this research.The original version of this article has been updated.

